# Negative feedback by NUR77/*Nr4a1* restrains B cell clonal dominance during early T-dependent immune responses

**DOI:** 10.1016/j.celrep.2021.109645

**Published:** 2021-08-31

**Authors:** Jeremy F. Brooks, Corey Tan, James L. Mueller, Kenta Hibiya, Ryosuke Hiwa, Vivasvan Vykunta, Julie Zikherman

**Affiliations:** 1 Division of Rheumatology, Rosalind Russell and Ephraim P. Engleman Rheumatology Research Center, Department of Medicine, University of California San Francisco, San Francisco, CA 94143, USA; 2 Lead contact

## Abstract

B cell clones compete for entry into and dominance within germinal centers (GCs), where the highest-affinity B cell receptors (BCRs) are selected. However, diverse and low-affinity B cells can enter and reside in GCs for extended periods. To reconcile these observations, we hypothesize that a negative feedback loop may operate within B cells to preferentially restrain high-affinity clones from monopolizing the early GC niche. Here, we report a role for the nuclear receptor NUR77/*Nr4a1* in this process. We show that NUR77 expression scales with antigen stimulation and restrains B cell expansion. Although NUR77 is dispensable for regulating GC size when GCs are elicited in a largely clonal manner, it serves to curb immunodominance under conditions where diverse clonal populations must compete for a constrained niche. We propose that this is important to preserve early clonal diversity in order to limit holes in the post-immune repertoire and to optimize GC selection.

## In brief

Brooks et al. define a new role for NUR77 in regulating clonal B cell competition during early humoral immune responses to immunization. The authors reveal that NUR77 is dispensable for germinal center size, but it serves to curb immunodominance when B cells must compete against each other into a limited niche.

## Graphical Abstract

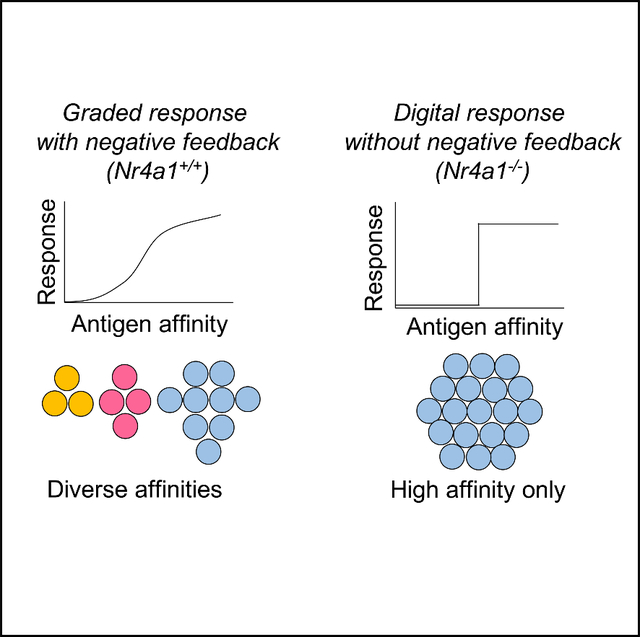

## INTRODUCTION

Germinal centers (GCs) nurture the diversification, proliferation, and selection of high-affinity B cell clones. After initial antigen encounter, up to 100 different B cell clones can seed an individual GC (Tas et al., 2016). This early clonal diversity is considered essential for driving optimal competition as the GC reaction evolves; excessive early dominance of a single high-affinity clone compromises optimal affinity maturation and risks inappropriately focusing the immune response on a non-neutralizing target, creating holes in the repertoire that pathogens could exploit (Abbott and Crotty, 2020; Le et al., 2008; Mesin et al., 2016). This provides a teleological rationale for the recruitment and preservation of clonally diverse B cells in the GC. Indeed, although B cells compete for limited T cell help prior to GC entry and within the GC itself (Schwickert et al., 2011; Shih et al., 2002; Woodruff et al., 2018), lower-affinity clones can nevertheless enter and reside in the GC for extended periods (Dal Porto et al., 1998, 2002; Kuraoka et al., 2016; Tas et al., 2016), suggesting that physiological mechanisms must exist to restrain high-affinity clones from monopolizing the early GC niche. Here, we test whether the orphan nuclear receptor NUR77/*Nr4a1* may restrain immunodominance in the early GC by mediating a negative feedback loop downstream of the B cell receptor (BCR).

NUR77 is rapidly upregulated by antigen receptor signaling and is thought to function as a ligand-independent transcription factor (Hazel et al., 1988; Milbrandt, 1988; Mittelstadt and DeFranco, 1993; Winoto and Littman, 2002). In T cells, NUR77 and its family members mediate thymic negative selection (Calnan et al., 1995; Liu et al., 1994; Woronicz et al., 1994), anergy (Liu et al., 2019), and exhaustion (Chen et al., 2019) and reinforce regulatory T cell (Treg) identity (Sekiya et al., 2013). Our lab has recently identified analogous roles for NUR77 in mediating B cell tolerance and limiting both T-dependent and T-independent B cell responses (Huizar et al., 2017; Tan et al., 2019, 2020). Through unbiased transcriptional profiling, we previously identified a novel set of targets repressed by NUR77 that are enriched for BCR-induced primary response genes (Tan et al., 2020). Interestingly, a subset of these target genes, including *Cd69*, *Cd86*, *Icam1*, *Ccl3*, and *Ccl4*, are important for B cell-T cell interaction. This led us to establish that NUR77 dampens the expansion of B cells when T cell help is limiting, in part, by restricting the acquisition of T cell help (Tan et al., 2020).

We previously took advantage of a fluorescent reporter of *Nr4a1* transcription (NUR77/*Nr4a1*-EGFP bacterial artificial chromosome [BAC] Tg) to show that its expression scales with both the dose and affinity of antigen stimulation, suggesting that negative regulation may likewise scale proportionately with BCR affinity (Tan et al., 2020; Zikherman et al., 2012). NUR77 expression is also enriched in light zone (LZ) B cells that are undergoing selection in the GC, suggesting that NUR77 may play an analogous negative regulatory role within the GC itself (Mueller et al., 2015). We therefore postulated that NUR77 mediates a negative feedback loop that tunes clonal diversity by disproportionately restraining the most strongly antigen (Ag)-stimulated B cell clones in a polyclonal repertoire. This would allow weakly stimulated, lower-affinity clones to participate in a humoral immune response and limit immunodominance of high-affinity clones. To test this, we designed a set of immunogens that enable us to systematically titrate relative immunodominance between a pair of competing B cell specificities. We find that NUR77 is dispensable for regulating GC size and composition when GCs are elicited in a largely clonal manner. Instead, NUR77 operates to restrain immunodominance under conditions where diverse clonal populations must compete for a constrained niche and does so, at least in part, in a B cell-intrinsic manner.

## RESULTS

### NUR77 regulates the expression of genes associated with T cell help and GC participation

**Figure 1.**
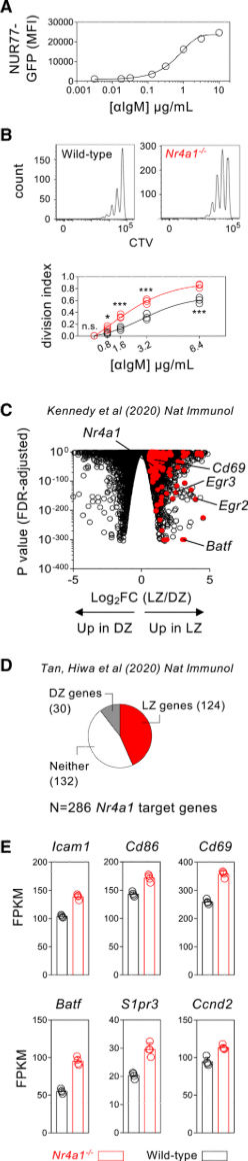
NUR77 negatively regulates expression of genes enriched in LZ GC B cells (A) Graph depicts mean fluorescence intensity (MFI) of NUR77/*Nr4a1*-GFP expression in reporter splenic B cells cultured for 24 h with varying concentrations of anti-IgM. (B) Cell Trace Violet (CTV)-loaded lymphocytes from WT or *Nr4a1*^*−/−*^ mice were cultured for 72 h with varying concentrations of anti-IgM. (Top) Representative histograms depict lymph node (LN) B cells stimulated with 3.2 μg/mL anti-IgM. (Bottom) Graph depicts division indices for LN B cells. In (A) and (B), each data point represents an individual mouse, and data are representative of at least three independent experiments ± SD. Data were modeled by non-linear regression, and statistical significance was assessed by a two-way ANOVA with a Holm-Sidak correction. (C) Genes that are associated with LZ or DZ GC B cells (p < 0.05) are depicted in a volcano plot (GEO: GSE133743; Kennedy et al., 2020). Overlaid in red are differentially expressed genes (DEGs) over-induced in *Nr4a1*^*−/−*^ B cells following 2 h of BCR stimulation (fold change > 1.2, p < 0.05) (GEO: GSE146747; Tan et al., 2020). (D) Pie chart depicts *Nr4a1* target genes (DEGs as defined above) that are enriched in either LZ or DZ B cells (from GEO: GSE133743 above) or do not appear to be associated with either population. (E) A consensus list of LZ-enriched genes was derived from independently published datasets (as described in STAR Methods). Graphs depict selected *Nr4a1* target genes that are enriched among consensus LZ GC B cells. Fragments per kilobase of transcript per million mapped reads (FPKM) from four biological replicates are plotted ± SD. Statistical analysis of published RNA sequencing (RNA-seq) datasets included in this figure was performed using EdgeR (Kennedy et al., 2020; Tan et al., 2020). All gene lists referred to in this figure are provided in Tables S1, S2, S3, S4, S5, and S6 for reference. *p < 0.05, ***p < 0.001. n.s., not significant.

We previously showed, using a fluorescent reporter of *Nr4a1* transcription (NUR77/*Nr4a1*-EGFP BAC Tg), that NUR77 expression scales with antigen receptor stimulation in B cells (Figure 1A) (Tan et al., 2020; Zikherman et al., 2012). We demonstrated that NUR77 limits both proliferation and survival of B cells following BCR stimulation (Figure 1B) (Tan et al., 2020; Tan et al., 2019). In addition to negative regulation of MYC expression, we also discovered that NUR77 reduces expression of genes that play a role in T cell-B cell interaction such as *Cd86* and that it concomitantly restrains early B cell expansion when T cell help is limiting (Tan et al., 2020). Indeed, not only is CD86 overinduced in the absence of NUR77 at early time points, but this difference increases with time and scales with intensity of BCR stimulation (Figures S1B and S1C). Because competition for T cell help is critical as B cell compete for entry into the GC and within the GC itself, we postulated that negative feedback mediated by NUR77 may also be relevant as T-dependent B cell responses evolve over time.

GCs harbor a geographic “division of labor” as B cells iteratively cycle between the dark zone (DZ) and the LZ. Clonal proliferation and antibody diversification occur in the DZ, while the LZ is the site where follicular dendritic cells (FDCs) display antigen for capture and presentation by competing B cell clones and where these clones vie for engagement of co-localized follicular helper T (Tfh) cells. Critical signals supplied by Tfh cells to LZ GC B cells in turn drive further clonal expansion in the DZ or selection into long-lived memory and plasma cell compartments (Mesin et al., 2016). Interestingly, publicly available datasets of GC B cell gene expression reveal enrichment of *Nr4a1*, as well as many of its target genes that we have previously identified and validated, among LZ GC B cells (Figure 1C) (Kennedy et al., 2020). Conversely, almost half of all genes that are repressed by NUR77 in B cells are also enriched for this LZ gene signature (Figure 1D; Tables S1, S2, and S3; Figure S1), including genes important for T-B interaction (*Icam1*, *Cd86*, *Slamf1*), GC B cell trafficking (*S1pr3*, *Cd6*9), and transcriptional regulators that govern GC fate (*Tbx21*, *Batf*, *Prdm1*, *Tet2*) (Tan et al., 2020). Importantly, many of these *Nr4a1* target genes could be identified across multiple independent datasets of LZ GC B cell gene expression (Figure 1E; Tables S4, S5, and S6) (Kennedy et al., 2020; Radtke and Bannard, 2019; Victora et al., 2010). These observations led us to propose that NUR77 may also impose a transcriptional negative feedback loop during LZ GC B cell selection.

### Competition in GCs that are largely clonal is not regulated by NUR77

**Figure 2.**
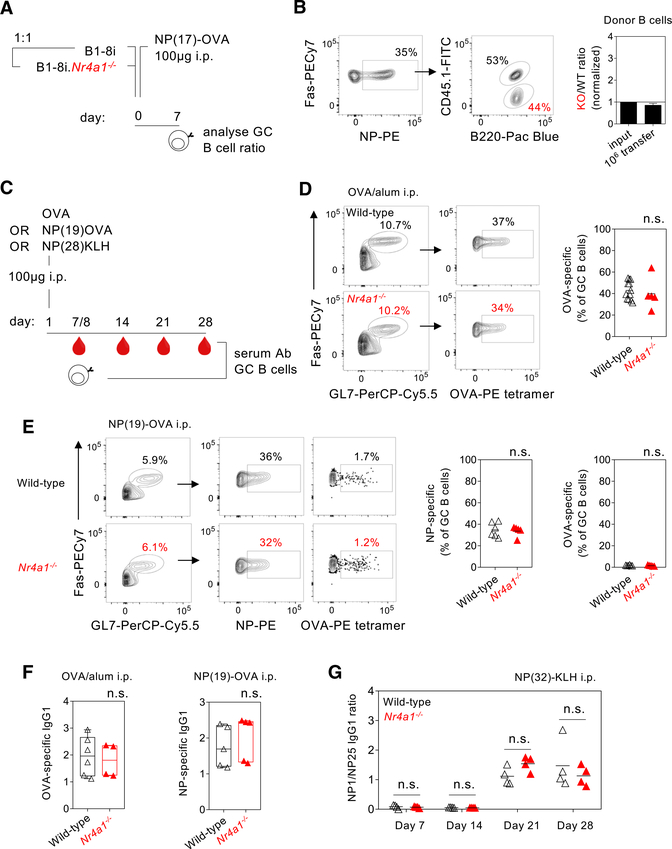
Competition in largely clonal GCs is not affected by NUR77 (A) Schematic of co-adoptive transfer of CD45.2^+^ B1–8i *Nr4a1*^*−/−*^ and CD45.1/2^+^ B1–8i *Nr4a*^*+/+*^ splenocytes into CD45.1^+^ host followed by immunization and analysis 7 days later. (B) (Left) Representative fluorescence-activated cell sorting (FACS) plots show NP^+^ B cells among Fas^+^GL7^+^ GCB cells with relative proportions of donor CD45.2^+^ *Nr4a1*^*−/−*^ and CD45.1/2^+^ *Nr4a*^*+/+*^ B cells following adoptive transfer of 10^6^ cells and immunization as in (A). (Right) Graph depicts ratio of *Nr4a1*^*−/−*^ relative to *Nr4a*^*+/+*^ NP-specific GCB cells, normalized to input. (C) Schematic of immunization conditions and analysis time points. (D) (Left) Splenocytes from OVA/alum-immunized *Nr4a1*^*−/−*^and wild-type (WT) control mice were stained with OVA tetramers at day 8. Representatives FACS plots depict OVA-binding Fas^+^GL7^+^ GC B cells. (Right) Graph depicts OVA-specific B cell frequency as a proportion of all GC B cells. (E) (Left) Splenocytes from NP19-OVA/alumimmunized *Nr4a1*^*−/−*^ and WT control mice were probed for NP or OVA binding at day 8. Representative FACS plots depict OVA-binding and NP-binding Fas^+^GL7^+^ GC B cells. (Right) NP-specific and OVA-specific B cell frequency as a proportion of all GC B cells. (F) (Left) OVA-specific IgG1 titers in day 8 serum from mice immunized with OVA/alum corresponding to (D). (Right) NP-specific IgG1 titers in day 8 serum from mice immunized with NP19-OVA/alum corresponding to (E). (G) Affinity of NP-specific antibodies was assessed in serum from *Nr4a1*^*−/−*^ and WT control mice immunized with NP32-KLH/alum at successive time points. Titer was assayed against plates coated with NP1-RSA and NP25-BSA and plotted as a ratio of NP1 titer divided by NP25 titer. Data are representative of at least two independent experiments (B and G) and show mean ± SD (B) or contain pooled data from at least two independent experiments (D–F) and show individual mice (D–G). Data were compared by an unpaired parametric t test (D–F) or two-way ANOVA with a Holm-Sidak correction (G). n.s., not significant.

To test the hypothesis that NUR77 negatively regulates GC B cell responses, we took advantage of widely used tools to comprehensively analyze GC size and clonality following immunization with various T-dependent immunogens. First, using a highly controlled setting, we adoptively co-transferred equal numbers of congenically marked (4-hydroxy-3-nitrophenyl)acetyl (NP)-specific B1–8i splenocytes from either *Nr4a1*^*+/+*^ or *Nr4a1*^*−/−*^ donors and then immunized recipients with a protein carrier highly conjugated with the hapten NP in order to assess T-dependent B cell responses directed exclusively against NP (NP17-ovalbumin [OVA]) (Figure 2A). Analysis of NP-specific B cells in GCs 7 or 13 days later revealed no competitive advantage for *Nr4a1*^*−/−*^ B cells in populating the GC (Figure 2B; Figure S2A). Transferring lower numbers of B1–8i splenocytes to encourage competition with endogenous NP-specific B cells similarly failed to reveal an impact of NUR77/*Nr4a1* (Figure S2B). It is possible that adoptive transfer of Ag-specific B cells might lead to expansion of Ag-specific T cells over time and thereby relax the stringency of competition between *Nr4a1*^*+/+*^ and *Nr4a1*^*−/−*^ donors. Therefore, we shifted our focus to the endogenous B cell repertoire. We next immunized *Nr4a1*^*−/−*^ mice and wild-type controls with either OVA, NP19-OVA, or NP28-keyhole limpet hemocyanin (KLH) in order to independently vary B cell and T cell epitopes. Immunogen-specific B cells and antibodies were then assessed at successive time points post-immunization (Figure 2C). Using OVA-labeled tetramers, which sensitively and specifically detect OVA-specific B cells (Brooks et al., 2018), we found no difference at day 8 in either the size of GCs or frequency of OVA-specific GC B cells following immunization with OVA (Figure 2D). Similarly, we found no difference in the frequency of NP-specific GC B cells following immunization with NP19-OVA (Figure 2E). Although OVA-specific B cells were detectable by tetramers in GCs elicited by NP19-OVA, they were extremely rare (<2% of total GC) (Figure 2E). In agreement with our analyses of GC composition, neither OVA-specific nor NP-specific IgG1 antibody responses differed between *Nr4a1*^*−/−*^ and wild-type mice (Figure 2F). We next generated competitive chimeras with a 1:1 mixture of congenically marked *Nr4a1*^*−/−*^ and wild-type donor bone marrow. After reconstitution, host chimeras were immunized with NP17-KLH, but we again found no specific advantage for endogenous NP-specific Nr4a1-deficient B cells (Figure S2D).

We next sought to probe selection of high-affinity antigen-specific B cells in the GC. In response to NP32-KLH, NP-specific affinity maturation assessed by ELISA across a time course was unaffected by NUR77 (Figure 2G). Finally, we took a complementary approach to probe affinity maturation; we again co-transferred *Nr4a1*^*−/−*^ B1–8i B cells and *Nr4a1*^*+/+*^ B1–8i B cells into a common host. We sequenced the heavy chain of NP-specific GC B cells at both early and late time points following immunization with NP17-OVA (Figure S2E). While we could clearly capture the expected increase in replacement mutations in the variable region and accumulation of the high-affinity W33L mutation with time, we found no difference between the two genotypes (Figure S2E). Collectively, our data reveal no role for NUR77 in modulating GC size or dynamics under the conditions tested.

### Reagents that titrate B cell competition reveal dynamic immunodominance in the GC

We noted that a feature common to all of the immunogens used in these experiments were the largely clonal GC responses they elicited. A combination of high precursor frequency, high immunogen avidity, and narrow mutational trajectory leads NP-specific B cells to reproducibly dominate GC reactions, regardless of their carrier proteins, when NP/hapten ratios are high (Abbott and Crotty, 2020). We wondered whether the high hapten density of immunogens used in these studies (NP17/19-OVA, NP17/32-KLH) might mask a role for NUR77 in regulating B cell clonal competition.

**Figure 3.**
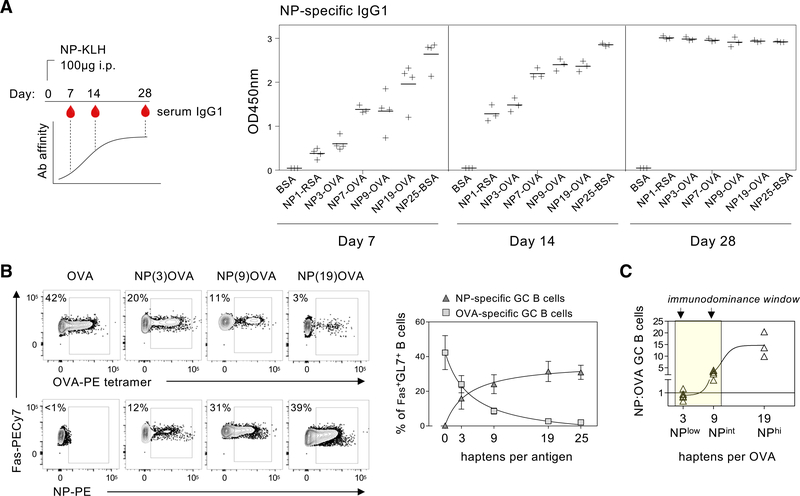
Hapten density titrates B cell clonal competition along a spectrum (A) (Left) Schematic shows WT mice immunized with NP32-KLH followed by serial bleeding via the lateral tail vein at successive time points (days 7, 14, and 28) post-immunization to obtain serum with increasing affinity for NP. (Right) Serum was analyzed for NP-specific IgG1 by coating ELISA plates with hapten antigens (10 μg/mL) conjugated with increasing numbers of hapten moieties or unconjugated BSA as a control. Each data point represents serum from an individual mouse, pooled from two independent experiments (except for NP7-OVA, a single experiment). (B) WT mice were immunized with OVA alone or haptenated OVA with increasing NP density. Splenocytes were probed 8 days later to detect antigen-specific GC B cells with NP-PE or OVA-PE tetramers. (Left) Representative plots are gated on Fas^+^GL7^+^ GC B cells and show NP-specific and OVA-specific GC B cells. (Right) Summary data comparing frequency of NP-specific and OVA-specific B cells within the germinal center at increasing hapten densities. (C) Calculated ratio of NP-specific B cells to OVA-specific B cells for each NP-OVA immunogen. Data are pooled (3–10 mice per group) and show mean ± SD (A and B). Plots show agonist-response three-parameter fit non-linear regression (B) or sigmoidal four-parameter fit non-linear regression (C).

We next sought to test the hypothesis that negative feedback by NUR77 might impact clonal diversity by preferentially restraining dominant B cell clones. Because NUR77 expression scales with BCR engagement, we reasoned that a NUR77-dependent negative feedback loop would operate specifically in a model system where avidity/affinity of BCR binding (rather than precursor frequency) drove clonal immunodominance. We searched for a reductionist approach to test this hypothesis and reasoned that reducing hapten density on carrier proteins would reduce avidity for hapten-specific B cells and might therefore permit recruitment of carrier protein-specific B cells and the development of more clonally diverse GCs. The corollary of this was that titration of hapten density could facilitate systematic modulation, and even inversion, of immunodominance because avidity is the basis for immunodominance in this system. To this end we generated NP-OVA reagents in-house by conjugating OVA to NP haptens at various molar ratios (Figure 3A). OVA was selected as the carrier protein because OVA-labeled B cell tetramers could reliably stain OVA-specific B cells (Figure S2C) and ELISA-based methods to measure serum OVA-specific immunoglobulin (Ig) were available (Figure 2F). This provided us with a tractable system to titrate and reliably detect clonal competition between NP and OVA-specific B cells within the GC. Based on absorbance at optical density 430 (OD_430_), we positioned three new haptenated OVA antigens (NP3-OVA, NP9-OVA, NP19-OVA) along a spectrum of other commercial hapten reagents (Figure S3A). We took two additional approaches to validate this hapten series *in vitro*. First, we assayed the binding of NP-specific antibodies derived from early (low-affinity) and late (high-affinity) stages of the humoral response to NP25-KLH (Figure 3A). As expected, based on hapten density, we found that hapten reagents were affinity sensing along a continuum at an early (day 7) but not late (day 28) time point when affinity maturation (and acquisition of the W33L mutation) was maximal. Second, we stimulated NP-specific B1–8i B cells *in vitro* with graded concentrations of our NP reagents and assessed the internalization of surface BCR via NP-phycoerythrin (PE) and λ1 co-stain. We found that the effective concentration required for at least 50% loss of surface BCR was reduced as the hapten density increased (Figure S3B). Taken together, these data position our NP-OVA conjugates on a spectrum alongside commercially sourced NP reagents.

We next sought to test the impact of these reagents on clonal competition by probing the GC compartment following immunization. As expected, GCs from mice immunized with OVA or NP19-OVA were dominated by either OVA- or NP-specific B cells, respectively (Figure 3B). Reducing the number of hapten moieties per OVA enabled a greater number of OVA-specific B cells to populate the GC (Figure 3B). Despite a profound competitive advantage for the NP specificity in the endogenous B cell repertoire, we found that NP immunodominance can be subverted in the GC when the number of hapten groups is dramatically reduced; NP3-OVA generated GCs that were dominated by OVA-specific rather than NP-specific B cells (Figure 3B). This was not due to masking of OVA epitopes by haptenation since OVA-specific antibodies could bind equally to NP3OVA and NP9OVA conjugates, as assessed by ELISA (Figure S3C). By mapping the ratio of NP- to OVA-specific GC B cells, our hapten series titrates clonal competition across a spectrum and identifies an inflection point where immunodominance shifts between NP-specific and OVA-specific B cells early after immunization (Figure 3C).

### NUR77 preferentially marks immunodominant B cells undergoing selection in the LZ

**Figure 4:**
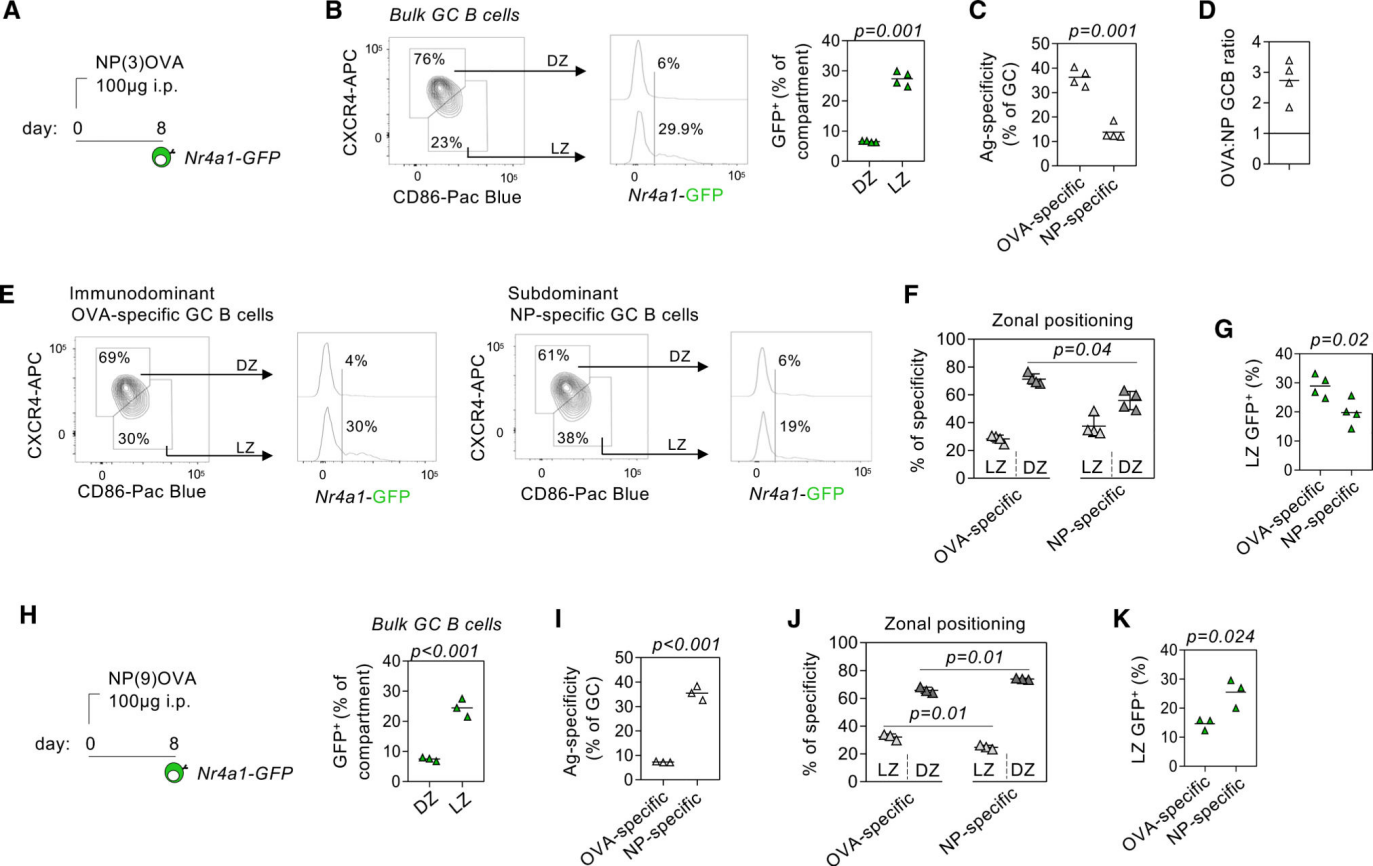
NUR77 expression preferentially marks immunodominant B cells in the GC LZ. (A) Schematic depicts immunization of NUR77/*Nr4a1*-GFP reporter mice with NP3-OVA. Spleens were harvested 8 days later to assess NUR77/*Nr4a1*-GFP among NP-specific and OVA-specific GC B cells. (B) (Left) Representative FACS plot showing total GC B cells gated to identify LZ (CD86^hi^CXCR4^lo^) and DZ (CD86^lo^CXCR4^hi^) compartments. Representative histogram shows GFP profile of LZ and DZ B cells. (Right) Graph depicts percentage of LZ and DZ compartments that are GFP^+^. (C) Graph depicts percentage of OVA-specific and NP-specific GC B cells. (D) Calculated ratio of OVA-specific to NP-specific GC B cells. Ratio >1 indicates OVA immunodominance. (E) Representative plots and histograms as in (B), but pre-gated on OVA-specific (left) or NP-specific (right) Fas^+^GL7^+^ GC B cells. (F) Graph depicts proportions of OVA-specific and NP-specific B cells resident in LZ or DZ compartments. (G) Graph depicts percentage of OVA-specific and NP-specific LZ GC B cells positive for NUR77/*Nr4a1*-GFP expression as gated in (E). (H–K) As in (A)–(C), (F), and (G) but using the NP(9)OVA reagent. Data are representative of two independent experiments (A–G) or a single experiment (H–K) and show individual mice ± SD. Data were compared by an unpaired parametric t test (B, C, G–I, and K) or ANOVA (F and J).

Because *Nr4a1* expression scales with the intensity of BCR ligation (Figure 1A) (Tan et al., 2020; Zikherman et al., 2012), it is possible that NUR77 acts to promote clonal diversity, rather than GC magnitude, by restraining the most strongly stimulated (immunodominant) B cells. We have previously used the NUR77/*Nr4a1*-EGFP reporter to show that NUR77 marks B cells undergoing selection in the LZ of the GC (Mueller et al., 2015). Indeed, the *Nr4a1* transcript is enriched among LZ GC B cells across published datasets (Tables S1, S2, S3, S4, S5, and S6). Here, we took advantage of these reporter mice and first asked whether NUR77/*Nr4a1*-EGFP expression was indeed enriched among immunodominant B cells. To this end, we elicited diverse but OVA-dominated GCs by immunizing *Nr4a1*-EGFP mice with the NP3-OVA reagent (Figure 4A). We recapitulated earlier observations that LZ B cells express high levels of NUR77/*Nr4a1-*EGFP reporter relative to DZ B cells (Figure 4B). We next confirmed that OVA- and NP-specific B cells populated the GC of reporter mice at frequencies and a ratio mirroring that observed in wild-type mice (Figures 4C and 4D). We next assessed the GFP profile among OVA- and NP-specific GC B cells and found that dominant OVA-specific B cells had substantially higher expression of GFP relative to NP-specific B cells (Figures 4 These results could be reproduced even when immunodominance was inverted using the NP9-OVA reagent (Figures 4H–4K). Because OVA and NP probes were both conjugated to PE fluorophores and analyzed in parallel tubes, differences in GFP were not attributable to differences in spectral overlap with the reporter. Interestingly, immunodominant B cells appeared to position in the DZ to a larger extent than subdominant B cells using either immunogen (Figures 4F and 4J), reminiscent of positively selected clones as described previously (Victora et al., 2010). These data suggest that NUR77 is upregulated among immunodominant GC B cells and support our hypothesis that this may serve as a mechanism to restrain such clones from monopolizing the GC niche.

### B cell immunodominance is modulated by NUR77

**Figure 5.**
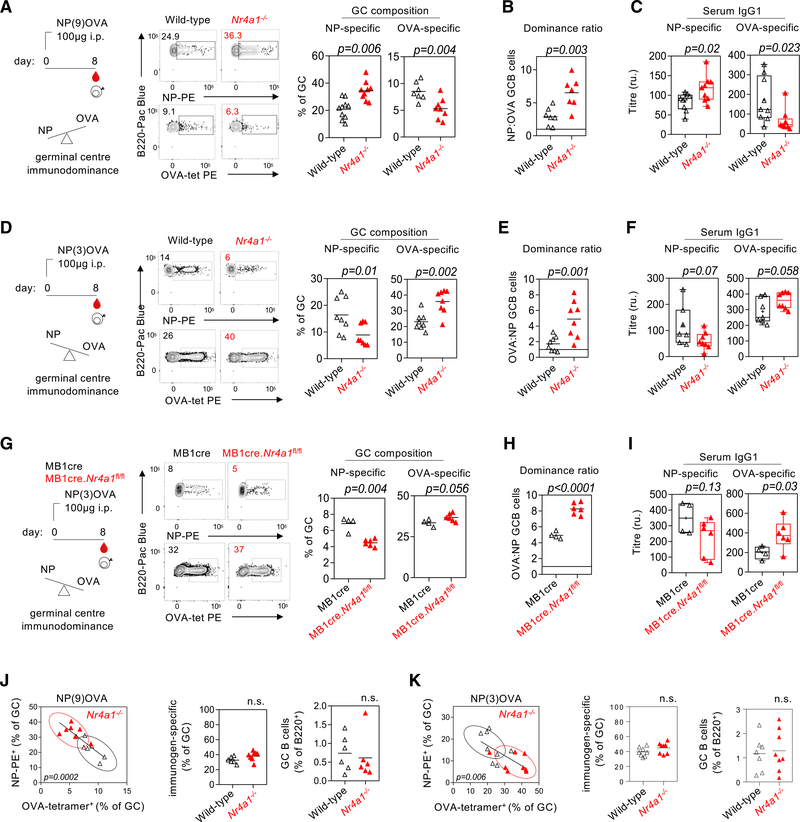
Loss of NUR77 skews clonal competition toward immunodominant B cells (A) (Left) Schematic of immunization with NP9-OVA followed by analysis of splenocytes 8 days later. NP-specific B cells are immunodominant relative to OVA-specific B cells under these conditions. (Middle) Representative FACS plots gated on Fas^+^GL7^+^ GC B cells show NP-PE and OVA-PE tetramer binding in WT and *Nr4a1*^*−/−*^ mice. (Right) Graph depicts NP-specific and OVA-specific B cells as a proportion of total GC B cells. (B) Graph depicts ratio of NP-specific B cells to OVA-specific GCB cells. Ratio >1 indicates NP immunodominance. (C) NP-specific and OVA-specific IgG1 titers were measured by ELISA at day 8. (D–F) as in (A)–(C) but for NP3-OVA. OVA-specific B cells are immunodominant relative to NP-specific B cells under these conditions. Ratio >1 indicates OVA immunodominance. (G–I) as in (D)–(F), but independently generated NP3-OVA immunogen was tested in MB1cre controls and MB1cre.*Nr4a1*^fl/fl^ mice. (J) (Left) NP-specific GC B cells plotted against OVA-specific B cells in the GC for individual mice 8 days following immunization with NP9-OVA and correspond to(A)–(C). (Middle) Graph depicts sum of NP-specific and OVA-specific B cells (i.e., immunogen-specific B cells) for individual mice as a proportion of all GC B cells. (Right) Graph depicts frequency of total GC B cells as a percentage of total B220^+^ B cells. (K) As in (J), but for NP3-OVA immunogen corresponding to (D)–(F). Data are pooled from three to five experiments (A–F, J, and K) or are representative of two independent experiments (G–I) and show individual mice. Data are compared by an unpaired parametric t test (A–I) or linear regression (J and K).

We next used our hapten reagents to formally test whether *Nr4a1* modulates inter-clonal B cell competition following T-dependent immunization *in vivo*. We first immunized mice with NP9-OVA, which elicits NP-dominant GCs (with a subdominant but sizeable OVA-binding population) 8 days following immunization (Figure 5A). We found that the genetic ablation of *Nr4a1* exacerbated immunodominance of NP-specific GC B cells relative to wild-type controls (Figure 5A). Concomitantly, subdominant OVA-specific B cells, as detected by tetramers, were rarer in GCs lacking NUR77 expression (Figure 5A). We next calculated a “dominance ratio,” in which the relative frequency of NP- to OVA-specific GC B cells in individual mice was computed, and found that loss of *Nr4a1* exaggerated NP immunodominance to the detriment of OVA-specific clones (Figure 5B). Importantly, we observed similar effects on NP-specific and OVA-specific IgG1 titers in *Nr4a1*^*−/−*^ mice at the same time point, implying that NUR77 functions to regulate clonal competition for entry into both the short-lived plasma cell (SLPC) compartment and GC (Figure 5C).

Because B cell precursor frequency is an important determinant of GC B cell composition (Abbott et al., 2018; Dosenovic et al., 2018), we sought to exclude the possibility that differences in NP-specific and OVA-specific B cell frequency could explain the effects on competition in *Nr4a1*^*−/−*^ GCs. By using a multi-tetramer approach (Brooks et al., 2018), we show that the frequency of OVA-specific B cells in naive, unimmunized *Nr4a1*^*−/−*^ mice did not differ from wild-type controls and is consistent with published findings (Taylor et al., 2012) (Figure S4A). Similarly, we found no impact of NUR77 expression on the development and frequency of NP-specific B cells in B1–8i transgenic mice (Figure S4B).

To definitively exclude any impact of B cell precursor frequency on our observations, and to establish that NUR77 regulates clonal dominance in response to a distinct immunogen, we took advantage of the NP3-OVA reagent in order to invert clonal immunodominance from NP-specific B cells to OVA-specific B cells. As predicted, OVA-specific B cell frequency was enhanced in GCs in the absence of NUR77 to the disadvantage of NP-specific B cells (Figures 5D–5F). In this setting, by computing the dominance ratio as the frequency of OVA- to NP-specific B cells (Figure 5E), we found that exaggerated immunodominance following NP3-OVA immunization in *Nr4a1*^*−/−*^ mice recapitulates our findings using NP9-OVA immunization. This result establishes that clonal immunodominance of NP-specific and OVA-specific B cells in response to immunization with NP9-OVA and NP3-OVA reagents is not attributable to clonal precursor frequency since the clonal dominance hierarchy is inverted.

### Regulation of dominance is B cell intrinsic

NUR77 expression can be detected in a variety of immune cell types at steady state and after activation. To isolate the contribution of NUR77 in B cells to modulation of immunodominance, we took advantage of B cell lineage-restricted deletion of *Nr4a1* using MB1cre (Tan et al., 2020). This cre is knocked in to the *Cd79a* locus and therefore efficiently deletes loxP-flanked sequences early during B cell development (Hobeika et al., 2006). We immunized MB1cre.*Nr4a1*^fl/fl^ and cre-only controls with NP3-OVA and assessed clonal composition of the GC as well as antibody titers 8 days later. Analogous to observations in mice with germline deletion of *Nr4a1*, we found that conditional loss of NUR77 in B cells also exacerbated immunodominance (Figures 5G–5I), although differences in the immunodominant fraction did not reach statistical significance (Figure 5G). Skewed immunodominance with NP9-OVA was not evident in the GC compartment, but we recapitulated suppression of subdominant OVA responses in the SLPC compartment of MB1cre.*Nr4a1*^fl/fl^ animals (Figures S4C–S4E), although this did not reach statistical significance. Taken together, these data suggest that the effect of NUR77 on clonal competition is partially, but not completely, B cell intrinsic.

### Regulation of dominance is independent of the T cell repertoire

We next sought to reproduce our observations in a different mouse line, with a different immunogen and cognate T cell repertoire. We selected the PE antigen for two reasons. First, the PE-specific immune response has been extensively studied and reagents to characterize the response by flow cytometry are robust (Pape et al., 2011). Second, PE-specific BCR gene usage has been mapped and reveals that a unique germline-encoded heavy chain variable region (V_H_1–81) gives rise to a high frequency of PE-specific B cells in mice carrying the *Ighb* but not *Igha* locus, and these have higher affinity for PE (Pape et al., 2018). We generated a tool to dissect the PE-specific immune response by crossing C57BL/6J (*Ighb*) mice with B6.IgH^a^ (*Igha*) mice, giving rise to IgH^a/b^ progeny. We reasoned that this would create a setting following immunization in which *Ighb*-derived PE-specific B cells would be immunodominant to their *Igha* counterparts within the same GC because of higher precursor frequency and affinity (Pape et al., 2011, 2018).

In naive IgH^a/b^ mice, PE-specific B cells were enriched by positive selection (Pape et al., 2011) and characterized. As expected, the PE-specific precursor pool, but not the total B cell pool, was disproportionately of IgH^b^ origin (Figures S5A–S5E). PE-specific IgH^b^ B cells from IgH^a/b^ mice were predominantly encoded by *IGHV1–81* as previously described, while IgH^a^ B cells were diverse (Pape et al., 2018) (Figure S5B). We then crossed B6.IgH^a^ to *Nr4a1*^*−/−*^ mice and immunized both IgH^a/b^ *Nr4a1*^*−/−*^ offspring and IgH^a/b^ controls with PE (Figures S5F–S5J). Importantly, precursor frequency of IgH^a^ and IgH^b^ PE-specific B cells were similar irrespective of NUR77 expression (Figure S5C). As predicted, IgM^b^ PE-specific B cells dominated GCs 8 days after immunization in both *Nr4a1*^*−/−*^ and wild-type mice (Figure S5H); indeed, the ratio of PE-binding IgM^b^/IgM^a^ cells in the GC was increased relative to the naive precursor ratios (Figures S5D and S5H). When the ratio of IgM^b^ to IgM^a^ PE-specific B cells was calculated, we again found that immunodominance was subtly skewed and increased in the absence of NUR77 (Figure S5J). These data are consistent with our observations using the NP-OVA system (Figure 5) and support a model in which NUR77 restrains clonal immunodominance and balances clonal competition into early GCs.

### Nr4a1 modulates clonal composition rather than GC magnitude

In all of our experiments, regardless of clonal distribution, we see that GC size is not regulated by *Nr4a1* (Figures 2 and 5). Indeed, comparing either the bulk GC B cell frequency or the sum of NP- and OVA-specific GC B cell frequencies (immunogen-specific) between wild-type and *Nr4a1*^*−/−*^ mice reveals no role for NUR77 in regulating the size of the GC niche (Figures 5J and 5K). Instead, NUR77 appears to modulate inter-clonal competition into a constrained niche. We conclude that, rather than controlling the total size of GC responses, NUR77 preferentially restrains immunodominant B cells to preserve clonal diversity.

## DISCUSSION

BCR affinity, immunogen avidity, and B cell precursor frequency all play critical roles in clonal competition during early T-dependent humoral immune responses (Abbott and Crotty, 2020; Abbott et al., 2018; Dosenovic et al., 2018; Kato et al., 2020; Paus et al., 2006; Schwickert et al., 2011; Shih et al., 2002; Woodruff et al., 2018). A stringently competitive environment within the GC subsequently drives further BCR affinity maturation. Homogenizing selection within different GCs in the same lymph node with rapid, synchronous kinetics has been repeatedly observed (Mesin et al., 2020; Tas et al., 2016), showing that one or a few clones will be spurred on to dominate the GC reaction by capturing a limited supply of antigen and monopolizing T cell help. Although this remains the central dogma of GC biology, there are notable exceptions. A mutation that is positively selected within one GC leading to dominance may not also lead to dominance in clonally related B cells sharing the same mutation in an adjacent GC (Tas et al., 2016). In the same lymph node, some GCs can be slower to select the fittest but eventually do so, and some GCs seemingly retain a highly diverse pool for extended periods without homogenizing (Tas et al., 2016). Even GC B cells that are of very low affinity or even non-specific for the immunogen (at least its native form) can enter and persist in the GC (Dal Porto et al., 1998, 2002; Kuraoka et al., 2016). While stochasticity in the evolution of individual GCs may explain some of this flexibility, we posit that negative feedback loops could also help to account for these observations and may restrain high-affinity B cell clones from monopolizing the GC niche too rapidly. Herein, we report proof-of-principle studies demonstrating that a negative feedback loop mediated by NUR77 in B cells can restrain clonal dominance at early time points in T-dependent humoral immune responses.

**Figure 6.**
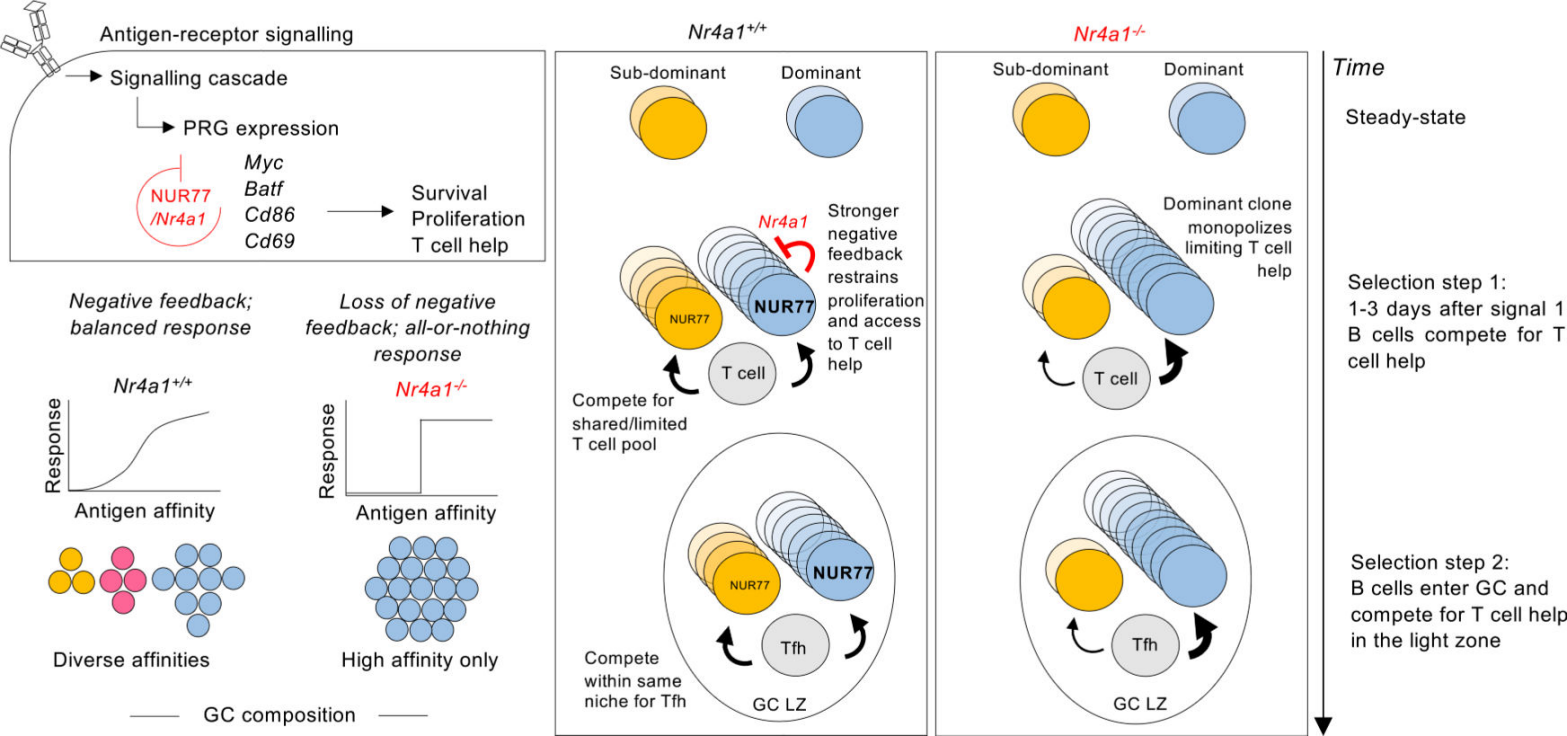
Model of negative feedback regulating GC composition (Top left) NUR77/*Nr4a1* is rapidly upregulated by BCR signaling and restrains expression of a subset of other primary response genes, imposing a negative feedback loop that serves to limit B cell expansion and recruitment of T cell help *when such help is limiting*. (Bottom left) Negative feedback produces a graded dose-response curve, while loss of negative feedback results in more digital, all-or-none responses. In the context of a polyclonal B cell response, such negative feedback preferentially restrains the highest affinity B cells from monopolizing limiting quantities of T cell help, enabling lower affinity clones to expand and populate the early GC niche. Our model predicts that this may serve to preserve clonal diversity during the course of the humoral immune response, while loss of negative feedback might be expected to enhance immunodominance. (Right) Upon immunization, both low-affinity subdominant and high-affinity dominant B cells become activated via BCR-antigen binding and upregulate NUR77 expression. NUR77 scales with the affinity/avidity of antigen binding, such that dominant B cells express higher levels of NUR77. NUR77 acts transcriptionally to restrain expression of target genes such as *Ccl3*, *Ccl4*, *Cd86*, and *Icam1*, which serve to recruit and engage T cell help. Dominant and subdominant B cells compete for the same finite, limiting T cell pool. Proportionate negative feedback in dominant B cells helps to restrain immunodominant clones and preserve participation of subdominant B cells in the early phase of the response. In contrast, in *Nr4a1*^*−/−*^ mice, immunodominant B cells are unrestrained and monopolize the available T cell help. We speculate that analogous competition among B cells for limiting Tfh cells may occur within the germinal center niche. Such competition is normally skewed in favor of high-affinity dominant clones, and this advantage is further exaggerated in the absence of *Nr4a1*, as unrestrained *Nr4a1*^*−/−*^ dominant B cells outcompete *Nr4a1*^*−/−*^ subdominant competitors in the light zone.

NUR77 is rapidly upregulated in naive B cells in response to BCR stimulation and limits the survival and expansion of B cells through several mechanisms, including repression of genes required for recruitment of T cell help (Moran et al., 2011; Tan et al., 2019, 2020; Zikherman et al., 2012). We have reported that NUR77 is also upregulated in GC B cells positioned in the LZ (Mueller et al., 2015), where antigen capture by the BCR and positive selection are thought to occur. In both acutely stimulated naive B cells and differentiated GC B cells, the expression of NUR77 reflects antigen-receptor signaling and scales with immunogen affinity/avidity (Figures 1A and 4) (Mueller et al., 2015; Tan et al., 2020). When T cell help is either absent or limiting, loss of NUR77 confers a competitive advantage to B cells (Tan et al., 2020). This advantage is due, in part, to upregulation of BATF and MYC in *Nr4a1*^*−/−*^ B cells following BCR stimulation and may also be driven by increased access to T cell help mediated by upregulation of CD86, ICAM1, and the T cell chemokines CCL3 and CCL4 (Tan et al., 2020). These findings supply a rationale for our hypothesis that clonally dominant B cells may be disproportionately restrained by NUR77 early in the immune response when T cell help is a limited common resource for which B cells must compete. Here, we report that, although total amplitude of the early GC response is unaffected, NUR77 regulates the clonal composition of GCs when B cells of varying affinity/avidity must compete for entry into a finite niche (presumably constrained by limiting T cell help). In the absence of NUR77, subdominant B cells are further outcompeted by dominant clones. This was true even when the clonal hierarchy was inverted by modifying the immunogen. Parallel analyses of antibody production at day 8 correlate with GC clonal competition at the same time point, implying that NUR77 regulates clonal competition prior to GC entry. Conversely, as a result of restraining dominant B cell clones, NUR77 expression facilitates participation of lower affinity, subdominant B cells in the evolving immune response and thereby may also promote clonal diversity, although this remains to be assessed directly as the GC reaction evolves. By virtue of modulating competitiveness for a limiting resource (T cell help), we propose that a partially cell-intrinsic role for NUR77 in B cells can impact clonal diversity (see model, Figure 6).

In these experiments, we deliberately chose to examine an early time point, as it provides a snapshot of GC composition that is interpretable within the context of our model system. In contrast, later time points involve acquisition of somatic mutations that alter affinity of individual B cell clones and create intra-clonal competition. Since NUR77 expression tracks with immunodominance inside the GC, it is possible that negative feedback in dominant B cells may partially restrain the clonal bursts that are responsible for collapse of later GC diversity as well (Tas et al., 2016). Further studies should test whether the effects of NUR77 early in the B cell response have lasting impact on retention and diversity at later time points in GCs, which were not assessed here. It is also possible that since the memory B cell pool is generated relatively early in the GC response (Mesin et al., 2020; Weisel et al., 2016), negative feedback may influence the clonality and magnitude of secondary responses to OVA or NP. Future studies that bypass early events using GC-specific deletion of NUR77, and thus can more directly test roles for NUR77 in GC B cell competition, will be of interest to isolate such roles and test this hypothesis.

One critical prediction from our model is that NUR77 promotes clonal diversity by restraining immunodominant B cell clones from monopolizing limited resources and outcompeting lower-affinity clones. Direct analysis of clonal diversity by Ig heavy chain variable region gene (IGHV) sequencing in response to a complex immunogen will be important to test this model in future work. A complementary approach could exploit Brainbow mice (Tas et al., 2016) in which clonal evolution can be traced through permanent multi-color fate mapping. The ability to track and quantify GC diversity at the cellular and *Ighv* sequence level using this system are well demonstrated (Mesin et al., 2020; Nowosad et al., 2020; Tas et al., 2016) and may facilitate future efforts to define how NUR77 impacts the rate of GC selection and collapse of clonal diversity.

Several lines of evidence suggest that the impact of NUR77 on clonal B cell competition is not mediated by a cell-intrinsic role for NUR77 in T cells. First, it has been reported by Qi and colleagues that NUR77 expression is dispensable for Tfh cell differentiation and function (Ma et al., 2015). Deletion of NUR77 from CD4^+^ T cells had no effect on the development of GC or production of class-switched antibodies (Ma et al., 2015). In agreement with our findings, Ma et al. (2015) also reported no change in the magnitude of GC in the context of germline deficiency for *Nr4a1*. Second, our observations were not dependent on the repertoire of T cells used to elicit GCs; NUR77 regulated clonal dominance regardless of whether help was solicited from either OVA-specific or PE-specific T cells. Third and most notably, although the magnitude was less pronounced, conditional ablation of *Nr4a1* in B cells using the MB1.cre line was sufficient to recapitulate effects on clonal immunodominance with NP(3)OVA, suggesting that they are at least partly mediated by NUR77 in B cells. However, the effects of NUR77 on clonal competition were only observed in the SLPC compartment of conditional knockout mice immunized with NP(9)OVA. Therefore, we cannot exclude a contribution of NUR77 in other cell types in coordinating early humoral responses.

Various modes of negative feedback governing GC B cell diversity have been proposed. Prior studies showed that pharmacologic inhibition of mTOR with rapamycin administration could facilitate GC participation by low-affinity flu-specific B cells that are typically outcompeted by dominant clones (Keating et al., 2013). Feedback by antibodies produced early in the response to infection or vaccination appears to modify GC composition over time by re-focusing competition; epitopes that elicited antibodies early may be shielded by antibody, providing the opportunity for other specificities to emerge in the GC toward additional epitopes. Both computational (Meyer-Hermann, 2019) and experimental (Angeletti et al., 2017; McNamara et al., 2020; Zhang et al., 2013) data support this concept. Of note, antibody-mediated negative feedback as a consequence of elevated serum antibody titers in *Nr4a1*^*−/−*^ mice are unlikely to account for the effects described herein, as they would be expected to restrain rather than exacerbate clonal immunodominance and lead to more diverse GCs. More recently, other pathways that may dynamically impact stringency of GC selection have been identified; for example, FcγRIIB is upregulated on FDC networks in the GC and appears to restrain clonal diversity in the GC, although the precise mechanism remains to be determined (van der Poel et al., 2019). It is speculated that its gradual upregulation in conjunction with GC formation may serve as a gatekeeper, while allowing a more diverse pool of B cells to enter early GC reactions when FcγRIIB expression is still low.

Why might negative feedback strategies serve an adaptive role in combatting pathogens? It is tempting to speculate that early clonal diversity evolved to facilitate competition and to maximize a broader mutational space that may be explored in the GC over time. Conversely, maximally efficient selection of a single dominant, high-affinity B cell clone can represent an Achilles heel of the immune system if it is non-neutralizing but outcompetes lower-affinity or rarer protective clones. Broadly neutralizing antibodies against pathogens such as HIV are difficult to elicit, in part because their germline precursors are rare and of low affinity (Cirelli et al., 2020). They also require considerable somatic mutation in the GC, which is hindered by competition with dominant but non-neutralizing epitopes. Thus, while substantial titers of high-affinity antibodies are the product of the GC reaction to HIV, these are at the expense of truly neutralizing BCRs. The poor recruitment and preservation of clonally diverse B cells hampers the generation of an effective HIV vaccine (Abbott and Crotty, 2020), underscoring the need to find innovative approaches that circumvent or at least modulate GC immunodominance. We propose that targeting negative feedback loops may constitute one such means to tune clonal composition. One strategy to achieve this goal may be to exploit the biology of NUR77 in B cells. Indeed, although NUR77 is thought to function as a constitutively active orphan nuclear receptor, a small-molecule agonist and antagonist ligands for NUR77/*Nr4a1* have been described (Chintharlapalli et al., 2005; Karki et al., 2020; Zhan et al., 2008, 2012). We propose that synthetic NUR77 ligands could serve as vaccine adjuvants to modulate clonal immunodominance.

In summary, our data provide evidence for a molecular pathway that operates in B cells to restrain immunodominance and preserve clonal diversity during early humoral immune responses. We speculate that this may serve an important function to limit holes in the post-immune repertoire that can be exploited by pathogens and to optimize affinity maturation of long-lived plasma cells.

### Limitations

There are several caveats to our study that are noted throughout and summarized below. First, the effects of NUR77 on clonal competition are due, at least in part, to a B cell-specific role for Nur77, as revealed by studies of mb1-cre *Nr4a1*^fl/fl^ animals. However, studies where *Nr4a1* is specifically deleted from B cells reveal a less profound effect on immunodominance than those performed in mice with germline deletion of *Nr4a1*. This suggests that Nur77 may also function in other cell types to restrain B cell clonal immunodominance. Second, we focused our study on early events in the humoral immune response following immunization, but we did not assess clonal competition and the role of Nur77 in regulation of GC clonal dynamics at later time points in the present study. This remains to be addressed formally in future work. Indeed, it is likely that antibody responses and GC composition at day 8 following immunization reflect events in activated B cells that occurred prior to GC seeding. We recently defined a mechanism by which Nur77 restrains B cell access to a limited supply of T cell help and propose that this may also regulate competition between dominant subdominant B cell clones during the evolution of humoral immune responses (Tan et al., 2020).

## STAR★METHODS

### RESOURCE AVAILABILITY

#### Lead contact

Correspondence should be addressed to the lead contact, Julie Zikherman (Julie.Zikherman@ucsf.edu).

#### Materials availability

Requests for resources and reagents should be directed to and will be fulfilled by the lead contact, Julie Zikherman (Julie.Zikherman@ucsf.edu).

#### Data and code availability

Processed data files for the RNA-seq analyses (corresponding to Figure 1) are provided in Tables S1, S2, S3, S4, S5, and S6. Raw RNA-seq data files have been deposited in public repositories associated with the following codes: Tan et al. (GEO: GSE146747), Kennedy et al. (GEO: GSE133743), Radtke and Bannard (GEO: GSE111419), Victora et al. (GEO: GSM589872). The paper does not report original code. Any additional information required to reanalyze the data reported in this paper is available from the lead contact upon request.

### EXPERIMENTAL MODEL AND SUBJECT DETAILS

All mice were housed in a specific pathogen-free facility at UCSF according to University and National Institutes of Health guidelines. All mice were backcrossed onto the C57BL/6J background for at least 6 generations. Male and female mice were randomly assigned to experiments between the ages of 6–14 weeks. Unless otherwise stated, C57BL6/J (CD45.2^+^) and BoyJ (CD45.1^+^) mice were used as wild-type experimental controls where appropriate.

#### NUR77/Nr4a1-GFP

BAC-Tg reporter mice that express GFP under the control of the *Nr4a1* regulatory region, originally generated by Gensat, distributed through MMRRC, and were previously described (Zikherman et al., 2012).

#### Nr4a1^fl/fl^

Offspring delete *Nr4a1* expression when crossed to mice bearing a conditional cre allele. *Nr4a1*^fl/fl^ mice were a gift from Pierre Chambon and Catherine Hedrick (Sekiya et al., 2013).

#### MB1.cre

MB1.cre mice harbor knock-in/knock-out with *Cre* introduced into the *Cd79a* gene. Cre expression in these mice drives deletion of a loxP-flanked allele in B cells early in development (Hobeika et al., 2006). Mice were sourced from the Jackson Laboratory.

#### MD4

Heavy and light chains encoding the HyHEL10 BCR are transgenically expressed in > 95% of B cells and endows high affinity binding to HEL (Goodnow et al., 1988). Mice were a gift from Jason Cyster and are available from the Jackson Laboratory.

#### Nr4a1^−/−^

Global deletion of *Nr4a1* generated by homologous recombination (Lee et al., 1995). Mice were sourced from the Jackson Laboratory.

#### B1–8i

Transgenically expresses a heavy chain that binds hapten with high affinity when paired with endogenous lambda light chain (Sonoda et al., 1997). ~5% of peripheral B cells bind hapten. Mice were sourced from the Jackson Laboratory.

#### C57BL/6J.IgH^a^

Mice were generated by crossing C56BL/6J (harboring 2 IgH^b^ alleles) mice to B6.IgH^a^ mice sourced from the Jackson Laboratory. B cells inherit heavy chains from both IgH^b^ and IgH^a^ origin but due to allelic exclusion, individual B cells can only rearrange and express heavy chains from one allotype. These can be reliably separated by congenic staining for b or a allotypes (e.g., IgM^a^ or IgM^b^).

#### MB1.cre Nr4a1^fl/fl^

MB1.cre*Nr4a1*^fl/fl^ were previously generated and validated in our laboratory through cross of the individual strains noted above (Tan et al., 2020). MB1.cre mice serve as the ‘wild-type’ experimental control experiments using MB1cre.*Nr4a1* mice.

### METHOD DETAILS

#### *In vitro* stimulation

Spleens and/or lymph nodes were pulverised through a 40μm cell strainer (Corning, #431750) to create a single-cell suspension. Red blood cells were lysed with ammonium chloride potassium (ACK) buffer. Either total To assess NUR77-GFP expression, 250,000 splenocytes were plated. To assess CD86 expression, 500,000 lymphocytes or purified B cells (Akadeum Life Sciences mouse B cell isolation kit, per manufacturer’s instructions) were plated. Cultures were stimulated with goat anti-mouse IgM F(ab’)2 (Jackson Immunoresearch) and analyzed at various time points (range 2–48 hours later). To assess proliferation, single cell suspensions of pooled (inguinal, axillary, brachial, cervical and mesenteric) lymph nodes were labeled with CellTrace Violet (CTV; Invitrogen) per the manufacturer’s instructions (except 5 × 10^6^ cells/ml rather than 1 × 10^6^ cells/ml). 125,000 cells were then plated and stimulated with anti-IgM F(ab’)2 and analyzed 72 hours later. For all *in vitro* experiments, live-dead exclusion was performed by incubating cells with LIVE/DEAD fixable near-IR dead cell stain kit (Invitrogen) according to manufacturer’s instructions, followed by surface staining. For experiments in which live B cells were sorted and sequenced, DAPI staining was used to identify cell viability.

#### Flow cytometry

Single cells were resuspended in fluorophore-labeled antibodies and incubated for 30 minutes on ice. All antibodies were used at a dilution of 1:200, were sourced from Tonbo Bioscience, BD Bioscience or Biolegend and include: B220-Pacific blue, B220-APC, CD19-BUV395, GL7-PerCPCy5.5, Fas-PECy7, CD23-Pacific Blue, λ1-biotin, IgMa-FITC, IgMb-biotin, CD45.2-PE, CD45.2-FITC, CD45.1-APC, CXCR4-APC, CD86-Pacific blue, IgD-eFluor780, CD21-PECy7, CD93-APC. All cytometry data was acquired by a Fortessa x20 (BD Bioscience) and data was analyzed using FlowJo (v10) software (Treestar Incorporated). Proliferation assessed via vital dye dilution was modeled using FlowJo.

#### Antigen-specific staining

##### NP-specific B cells

To stain for NP-specific B cells, NP25-PE or NP23-PE was used at 1:200 dilution (LGC Biosearch Technologies).

##### OVA-specific B cells

To stain for OVA-specific B cells, OVA (Sigma) was biotinylated (Invitrogen) to a ratio of 1 biotin molecule per OVA as previously described (Brooks et al., 2018). Tetramers were then constructed fresh on the day of use by mixing biotinylated OVA with streptavidin-PE or streptavidin-APC at a 4.1:1 molar ratio for 2 hours on ice. Tetramers were diluted 1:100–1:200 for staining.

##### PE-specific B cells

To stain for PE-specific B cells from naive mice, single cell suspensions of splenocytes were stained with 1μg PE and subjected to anti-PE magnetic enrichment as previously described (Pape et al., 2011). The enriched fraction was then eluted and stained for surface markers as described above. PE-specific B cells analyzed following immunization were not pre-enriched prior to analysis.

#### Radiation bone marrow chimeras

Recipient CD45.1 BoyJ mice were conditioned with two doses of 5.3Gy, 4 hours apart, and then injected i.v. with 10^6^ mixed bone marrow cells from CD45.1/2 *Nr4a1*^*+/+*^ and CD45.2 *Nr4a1*^*−/−*^ donors. Mice were rested for at least 10 weeks to allow bone marrow reconstitution prior to immunization.

#### Adoptive cell transfers

For adoptive cell transfers, spleens from B1–8i Tg mice were harvested into single cell suspensions. 10^4^, 10^5^, or 10^6^ splenocytes from CD45.1/2 B1–8i *Nr4a1*^*+/+*^ and CD45.2 B1–8i *Nr4a1*^*−/−*^ mice mixed in a 1:1 ratio were then transferred to recipient CD45.2 BoyJ mice i.v in 200μL PBS.

#### Immunizations

Immunogens were admixed 1:1 with Alhydrogel 1% adjuvant (Accurate Chemical and Scientific Corp.) and injected i.p. at a dose of 100μg/mouse. NP17-OVA, NP17-KLH, NP28-KLH and NP32-KLH were purchased from LGC Biosearch. OVA was purchased from Sigma. All other NP conjugates were made in-house.

#### NP conjugation

NP-OSu linker (LGC Biosearch) was dissolved in DMSO to 100mM. OVA was dissolved in carbonate buffer (pH 8.0) to 10mg/mL. Linker and OVA (in 5mL) were then mixed at various molar ratios (NPlow (3) – equal molar ratio; NPmed (7,9) – 5-fold excess linker; NPhigh (19) – 10-fold linker excess) overnight at room temperate with continuous agitation. Conjugates were centrifuged to pellet precipitated protein and soluble antigen was removed for further processing. Excess linker from the soluble fraction was removed by 3 rounds of dialysis in 10kDa snakeskin (ThermoFisher) against PBS at room temperature. Protein concentration was measured by nanodrop and NP-conjugates were stored at 4°C. Hapten ratio was determined by calculating the molar ratio of protein (A280nm) to hapten (A430nm) using known extinction coefficients (NP_430_ = 4230, OVA_280_ = 30590).

#### ELISA

Serum was harvested from blood collected by lateral tail vein sampling or cardiac puncture postmortem. OVA-specific and NP-specific IgG1 ELISA was performed as described (Brooks et al., 2020; Tan et al., 2020). Briefly, ELISA plates were coated with NP conjugates (NP1-RSA, NP25-BSA, NP10-BSA all from LGC Biosearch; NP3-OVA, NP7-OVA, NP9-OVA and NP19-OVA were conjugated in-house as described above), OVA (Sigma Aldrich), BSA (Research Products International) or HEL antigen (10μg/mL; Sigma Aldrich), samples were added, and HRP-labeled anti-IgG1 antibodies (Southern Biotech) were used to detect plate-bound IgG1. Plates were developed with slow kinetic form TMB (Sigma Aldrich) and stopped with 1N sulfuric acid. Absorbance was measured at 450nm using spectrophotometer (SpectraMax M5, Molecular Devices). Relative titers were interpolated from standard curves generated using samples with known high titers of antigen-specific antibody.

#### IgH sequencing

For PE-specific B cells, universal IgH sequencing was performed as described (Pape et al., 2018). Briefly, B cells of interest were sorted by FACS (Aria II, BD Bioscience) into Trizol (Life Technologies) and RNA was extracted per protocol. cDNA was synthesized and PCR amplified with Onestep RT-PCR kit (QIAGEN). Ighv alleles were amplified using a common variable region primer msVHE (5′GGGAATTCGAGGTGCAGCTGCAGGAGTCTGG3′) and a specific constant chain primer for either IgM (5′GATACCCTGG ATGACTTCAGTGTTG3′) or IgG (5′CACACCGCTGGACAGGG3′) (Pape et al., 2018). For cloning of NP-specific GC B cell HC, the same approach was used except amplification was achieved using a distinct forward primer: VH186.2_EXT F (5′GATGGAGCTGTATCATGCTCTTCTTGGCAG3′). The same reverse constant chain primers were used as above. The cDNA was then run on a 2% agarose gel, and extracted with the Qiaquick Gel Extraction kit (QIAGEN). cDNA was then inserted into the PCR-2.1 Topo vector through the use of the Topo-TA kit (Invitrogen), and transformed into Top10 competent *E. coli* (Invitrogen) via heat shock. Colonies were expanded at 37°C overnight on ampicillin plates to select for transformants and were then picked and PCR amplified using M13 primers from the Topo-TA kit. The amplified DNA was then treated with recombinant Shrimp Alkaline Phosphatase and Exonuclease 1 (New England Biolabs), and sent to ELIM Biopharm for sequencing. The returned PE-specific sequences were then clipped, the vector sequence was trimmed, and subsequently uploaded to IMGT_V-QUEST (IGMT.org) for alignment and heavy chain identification. For NP-specific sequences, alignment with germline reference was performed to identify coding and non-coding SHM.

#### Analysis of transcriptional datasets

Transcripts enriched in *Nr4a1*^*−/−*^ B cells relative to WT following 2 hr BCR stimulation were identified from a recently published RNaseq dataset (Tan et al., 2020) – GEO: GSE146747. Differentially expressd genes (DEG) were previously assessed via EdgeR analysis. To identify NUR77/*Nr4a1* target genes, a threshold of > 1.2 fold enrichment and p < 0.05 was applied (Table S1). Additionally, three independent analyses of DEG between LZ and DZ GC B cells were retrieved: (Kennedy et al., 2020) – GEO: GSE133743; (Radtke and Bannard, 2019) – GEO: GSE111419; (Victora et al., 2010) – GEO: GSM589872. RNaseq data from GEO: GSE133743 (Kennedy et al., 2020) was used as a template to establish an LZ “gene signature”; genes enriched in LZ and DZ (excluding ‘gray zone’) were identified as > 1.5-fold upregulated relative to each other with p < 0.05 via prior analysis using EdgeR (Table S2). *Nr4a1* target genes were compared to both of these LZ and DZ gene signatures and presented in Figure 1 and Table S3). DEG enriched in LZ > 1.33 fold (Victora et al., 2010) and > 1.5 fold (Radtke and Bannard, 2019) were then identified (Tables S4 and S5), and LZ DEG that appeared in at least 2 of 3 datasets were classified as “LZ consensus genes” (Table S6). *Nr4a1* target genes as defined above were then referenced to this consensus list to identify putative *Nr4a1* targets in LZ GC B cells (Table S6).

### QUANTIFICATION AND STATISTICAL ANALYSIS

All data were analyzed in GraphPad Prism (v7.0, GraphPad Software Inc.). For comparison of two groups, data were compared by two-tailed parametric t test where appropriate unless otherwise stated (in the figure legends). For comparison of more than two groups over time, analysis was performed using multiple t tests corrected by Holm-Sidak posthoc analyses. Curve fit analyses used non-linear regression (4-parameter agonist-response function in Prism Software). P values shown graphically denote the following: *p < 0.05, **p < 0.01, **p < 0.001. Data show individual mice ± SD unless otherwise stated. The number of mice and replicates can be found within the figure legends. Adjusted P values for RNA-seq data were extracted from the analyzed datasets deposited under the GEO accession codes provided in the supplemental items section. Information for how raw RNA-seq data was treated and analyzed can be found in the corresponding citations. Information for how the analyzed RNA-seq data was further analyzed for this manuscript can be found under the STAR Methods sections called *Analysis of Transcriptional Datasets*.

## Supplementary Material

1

2

3

4

5

6

7

## Figures and Tables

**KEY RESOURCES TABLE:** 

REAGENT or RESOURCE	SOURCE	IDENTIFIER
Antibodies		

Rat Anti-Mouse CD45R (B220) Pacific Blue, clone RA3–6B2	BD PharMingen	Cat#558108; RRID: AB_397031
Rat Anti-Mouse CD45R (B220) AF647, clone RA3–6B2	BD PharMingen	Cat#557683; RRID: AB_396793
Rat Anti-Mouse CD19 BUV395, clone 1D3	BD PharMingen	Cat#563557; RRID: AB_2722495
Mouse Anti-Mouse CD45.2 PE, clone 104	BD PharMingen	Cat#560695; RRID: AB_1727493
Mouse Anti-Mouse CD45.2 FITC, clone 104	BD PharMingen	Cat#553772; RRID: AB_395041
Rat Anti-Mouse GL7 PerCPCy5.5, clone GL7	Biolegend	Cat#144610; RRID: AB_2562979
Hamster Anti-Mouse CD95 (Fas) PECy7, clone Jo2	BD PharMingen	Cat#557653; RRID: AB_396768
Rat Anti-Mouse CD23 Pacific Blue, clone B3B4	Biolegend	Cat#101616; RRID: AB_2103306
Rat Anti-Mouse Lambda 1, clone R11–153	BD PharMingen	Cat#553431; RRID: AB_394851
Mouse Anti-Mouse IgM^a^ FITC, clone MA-69	Biolegend	Cat#408606; RRID: AB_940541
Mouse Anti-Mouse IgM^b^ Biotin, clone AF6–78	Biolegend	Cat#406204; RRID: AB_315037
Mouse Anti-Mouse CD45.1 APC, clone A20	eBioscience	Cat#17–0453-82; RRID: AB_469398
Rat Anti-Mouse CXCR4 APC, clone L276F12	Biolegend	Cat#146508; RRID: AB_2562785
Rat Anti-Mouse CD86 Pacific Blue, clone GL-1	Biolegend	Cat#105022; RRID: AB_493466
Rat Anti-Mouse IgD APC-eFluor 780, clone 11–26c	eBioscience	Cat#47–5993-82; RRID: AB_2573994
Goat Anti-Mouse IgG1 HRP	Southern Biotech	Cat#1070–05; RRID: AB_2650509
Streptavidin FITC	Biolegend	Cat#405202
Streptavidin Pacific Blue	Life Technologies	Cat#S11222
Streptavidin PE	BD PharMingen	Cat#554061
Streptavidin APC	Biolegend	Cat#405207

Chemicals, peptides, and recombinant proteins		

NP-OSu	LGC Biosearch Technologies	Cat#N-1010–100
AffiniPure F(Ab’)_2_ Fragment Goat Anti-Mouse IgM, μ Chain Specific	Jackson Immuno Research	Cat#115–006-020; RRID: AB_2338469
EZ-Link Sulfo-NHS-LC-biotin	ThermoFisher	Cat#21335
Lysozyme from chicken egg white	Sigma	Cat#L6876
Albumin from chicken egg white (OVA)	Sigma	Cat#A5503
NP-BSA	LGC Biosearch Technologies	Cat#N-5050H-10
NP-KLH	LGC Biosearch Technologies	Cat#N-5060–5
NP-OVA	LGC Biosearch Technologies	Cat#N-5051–10
NP-RSA	LGC Biosearch Technologies	Cat#N-5051–10
NP-PE	LGC Biosearch Technologies	Cat#N-5070–1
Alhydrogel adjuvant 2%	InvivoGen	Cat#vac-alu-250
Freund’s Adjuvant, Complete	Sigma	Cat#F5881
Critical commercial assays		
LIVE/DEAD Fixable Near-IR Dead cell stain kit	ThermoFisher	Cat#L10119
Anti-PE Microbeads	Miltenyi Biotec	Cat#130–048-801
Mouse B cell isolation kit	Akadeum Life Sciences	Cat#22210–110
Qiaquick Gel Extraction Kit	QIAGEN	Cat#28506
Topo-TA Cloning Kit for Sequencing, with One Shot TOP10 Chemically Competent *E.coli*	ThermoFisher	Cat #K457501
Cell trace violet (CTV) Proliferation Kit, for Flow Cytometry	ThermoFisher	Cat #34557
Pierce biotin quantification kit	ThermoFisher	Cat #28005

Deposited data		

RNaseq of wild-type and *Nr4a1*^*−/−*^ B cells	Tan et al., 2020	GEO: GSE146747
RNaseq of light zone GC B cells	Kennedy et al., 2020	GEO: GSE133743
RNaseq of light zone GC B cells	Radtke and Bannard, 2019	GEO: GSE111419
Microarray of light zone GC B cells	Victora et al., 2010	GEO: GSM589872
Experimental models: Organisms/strains		
Mouse: NUR77/Nr4a1-GFP	MMRRC: 012015-UCD	PMID: 22902503
Mouse: Nr4a1fl/fl	Pierre Chambon; Catherine Hedrick	PMID: 23334790
Mouse: MB1.cre	Jackson labs: Stock No: 020505	PMID: 16940357
Mouse: MD4	Jackson labs: Stock No: 002595	PMID: 3261841
Mouse: *Nr4a1*^*−/−*^	Jackson labs: Stock No: 006187	PMID: 7624755
Mouse: B1–8i	Jackson labs: Stock No: 012642	PMID: 9075923
Mouse: C57BL6/J.IgHa	Jackson labs: Stock No: 001317	N/A
Mouse: MB1cre.Nr4a1fl/fl	Bred using above	PMID: 32868928
Oligonucleotides		
msVHE: 5′GGGAATTCGAGGTGCAGCTGCAGGAGTCTGG3′	N/A	PMID: 29884459
IgM: 5′GATACCCTGGATGACTTCAGTGTTG3′	N/A	PMID: 29884459
IgG: 5′CACACCGCTGGACAGGG3′	N/A	PMID: 29884459
VH186.2: 5′GATGGAGCTGTATCATGCTCTTCTTGGCAG3′	N/A	PMID: 26915335

Software and algorithms		

GraphPad Prism	GraphPad Software Inc.	https://www.graphpad.com/scientific-software/prism/
FlowJo	TreeStar Inc.	https://www.graphpad.com/scientific-software/prism/
IMGT_V-QUEST	IMGT http://www.imgt.org/download/V-QUEST/	PMID: 18503082
CodonCode Aligner	CodonCode	https://www.codoncode.com/aligner/
BD FACSDiva	BD Biosciences	https://www.bdbiosciences.com/en-us/products/software/instrumentsoftware/bd-facsdiva-software
ClustVis	BIIT ClustVis	PMID: 25969447

## Highlights

- NUR77/*Nr4a1* expression scales with antigen stimulation in B cells
- NUR77 represses a transcriptional program associated with the GC light zone
- NUR77 does not regulate GC size in response to immunization
- NUR77 regulates B cell competition to limit clonal immunodominance
