# Supplementary material for: Negative feedback by NUR77/Nr4a1 restrains B cell clonal dominance during early T-dependent immune responses
Source: Cell Rep. Author manuscript; Available in PMC 2021 Nov 3. (PMC8564879; doi:10.1016/j.celrep.2021.109645)
Supplement: 1 [file NIHMS1737340-supplement-1.pdf]

Cell Reports, Volume *36*

Supplemental information

Negative feedback by NUR77/*Nr4a1* restrains B cell clonal dominance during early T-dependent immune responses

Jeremy F. Brooks, Corey Tan, James L. Mueller, Kenta Hibiya, Ryosuke Hiwa, Vivasvan Vykunta, and Julie Zikherman

Supplementary Fig 1. NUR77/Nr4a1 regulates gene expression, Related to main figure 1.

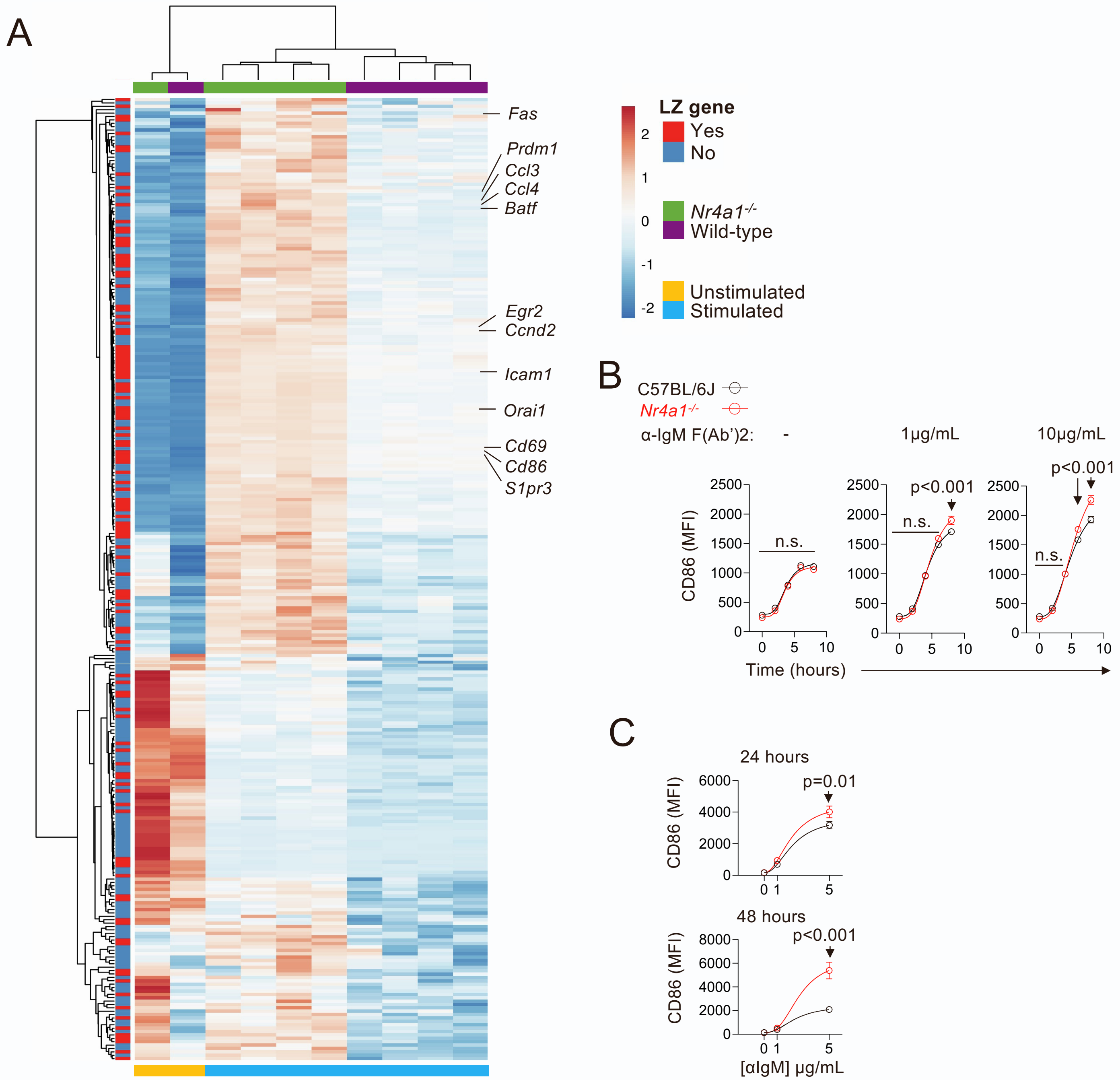

A, Heatmap of DEG over-induced in *Nr4a1*$^{-/-}$ B cells following 2 hr BCR stimulation (fold change > 1.2 and p<0.05), corresponding to main Figure 1D (GSE146747; Tan et al., 2020). Annotated LZ genes correspond to main Figure 1C, D and Supplemental Data 1-Tables 1-3 (GSE133743; Kennedy et al., 2020). Heatmap was assembled using the ClustVis online tool and annotated for LZ genes (left-hand side). Data show independent biological replicates (n=4 per genotype for stimulated, n=1 per genotype from unstimulated) from GSE146747. **B,** Lymphocytes from CD45.1 wild-type and CD45.2 *Nr4a1-/-* mice were co-cultured with varying doses of anti-IgM and surface CD86 expression on B220+ cells was measured by flow cytometry up to 8 hours post-stimulation. **C,** B cells were purified by bench-top negative selection from spleen and lymph node of wild-type and *Nr4a1-/-* mice and cultured independently with varying doses of anti-IgM. CD86 expression on B220+ cells was measured at 24 hours (top) or 48 hours (bottom). Curves were modelled using non-linear regression and datapoints were compared by multiple T-tests corrected by Holm-Sidak post-hoc analysis. Data shows mean of 3 mice ± SEM from a single experiment, representative of at least 4 experiments.

Supplementary Fig 2. NUR77/Nr4a1 has no effect on clonal GCs, Related to main figure 2.

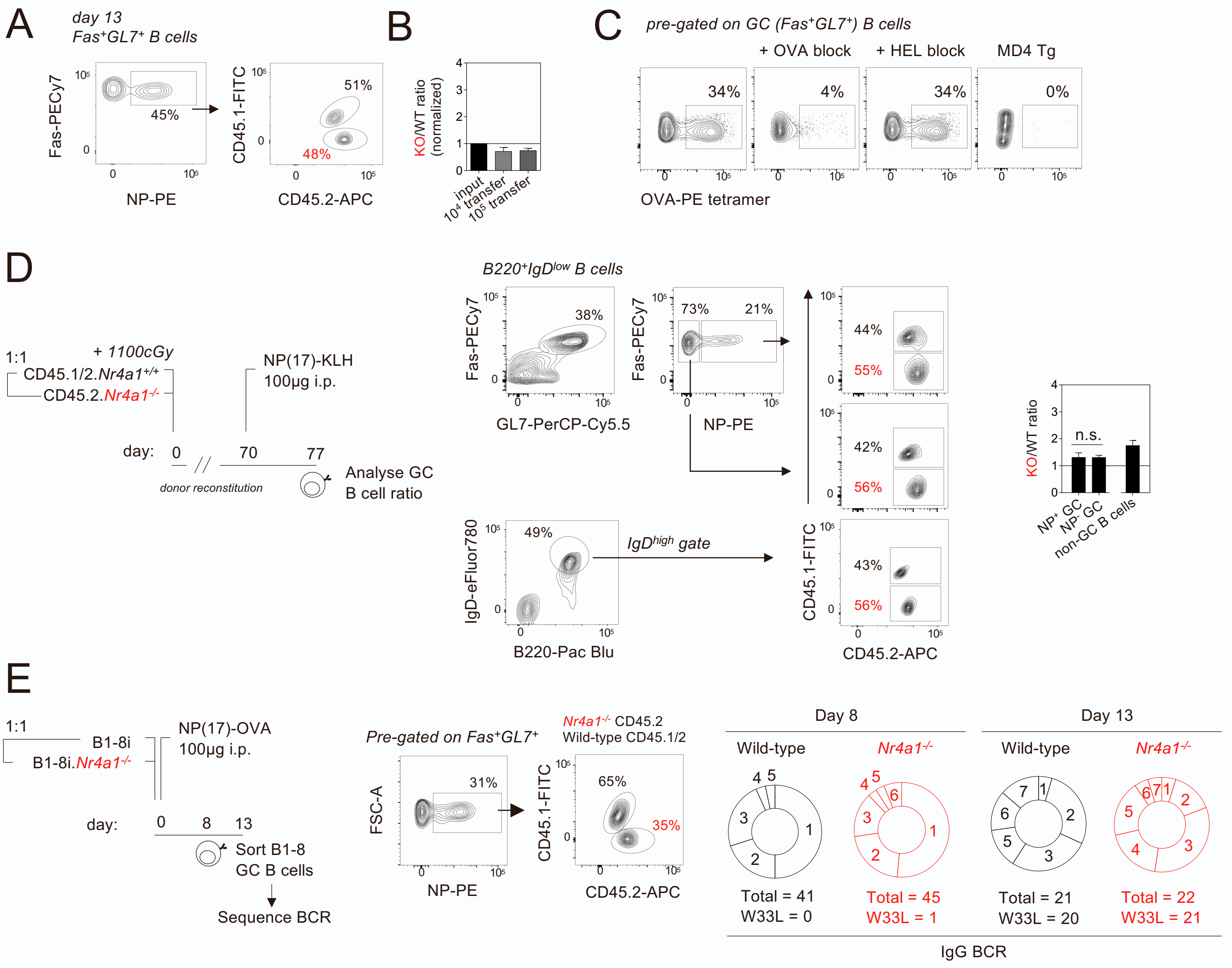

A, Representative FACS plots showing NP-specific GC B cells 13 days following co-adoptive transfer of CD45.2+ B1-8i *Nr4a*$^{-/-}$ and CD45.1/2+ B1-8i *Nr4a*$^{+/+}$ splenocytes into CD45.1+ host and immunization with NP17-OVA. **B,** Experiment performed as in Figure 2A,B, except either 10^4 or 10^5 donor splenocytes (containing a 1:1 mixture of CD45.2+ B1-8i *Nr4a1*$^{-/-}$ and CD45.1/2+ B1-8i *Nr4a*$^{+/+}$) were adoptively transferred prior to immunization. Graph depicts ratio of donor genotypes identified among NP-specific GC B cells after 7 days, normalized to input. **C,** Representative FACS plots of GC B cells 8 days following immunization with OVA/alum. Spleens were probed with OVA-PE tetramers. As controls, cells were pre-blocked with OVA or the irrelevant protein HEL, and in parallel, HEL-specific Ig-transgenic MD4 B cells were also stained with OVA-PE tetramers. **D,** *(left)* schematic depicts generation of competitive radiation chimera with 1:1 mixture of donor CD45.2+ *Nr4a1*$^{-/-}$ and CD45.1/2 *Nr4a1*$^{+/+}$ donor bone marrow transplanted into CD45.1+ host. Following 10 weeks of reconstitution, recipients were immunized with NP17-KLH and analysed 7 days later. *(middle)* representative FACS plots depict gating scheme to identify NP+ and NP- GC B cells or naïve follicular IgDhi B cells of each donor genotype, wild-type (CD45.1/2) and *Nr4a1*$^{-/-}$ (CD45.2) cells. *(right)* graph depicts ratio of *Nr4a1*$^{-/-}$ B cells to wild-type B cells within each gate. **E,** *(left)* Schematic depicts co-adoptive transfer of CD45.2+ B1-8i *Nr4a1*$^{-/-}$ and CD45.1/2+ B1-8i *Nr4a*$^{+/+}$ splenocytes into CD45.1+ hosts. Recipients were immunized with NP17-OVA and B1-8i donor GC B cells were sorted either 7 or 13 days later for heavy chain sequencing. *(middle)* representative plots show gating scheme to identify NP-specific GC B cells of donor wild-type (CD45.1/2) and *Nr4a1*$^{-/-}$ (CD45.2) origin at day 8. *(right)* Pie graphs depicting the number of replacement mutations in the VDJ region of the VH186.2 heavy chain. Below are listed total number of sequences analysed as well as number harbouring high affinity W33L mutation. Data are from single experiments with n=3-4 mice (A, B, D) or a single mouse (E). n.s., non-significant.

Supplementary Fig 3. Validation of new NP-OVA conjugates, Related to main figure 3.

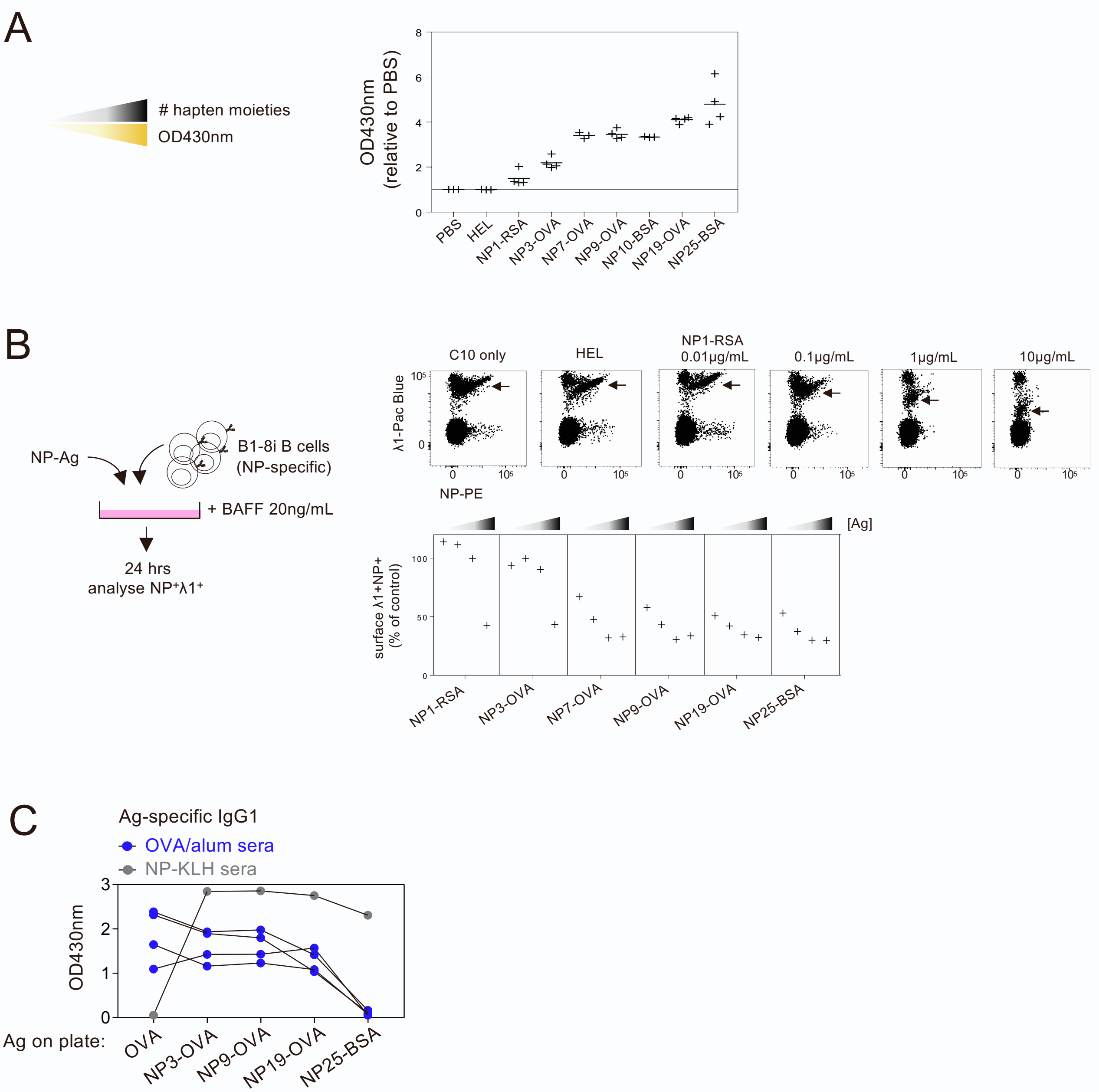

A, *(left)* Hapten density is measured spectrophotometrically by absorbance at OD430nm. *(right)* Antigens (1mg/mL) conjugated with increasing numbers of hapten moieties, as well as unconjugated control (sHEL) and PBS, were analysed for absorbance at 430nm. Each datapoint represents a technical replicate, data are pooled from two independent analyses. **B,** *(left)* Schematic depicts 24 hr culture of splenocytes from B1-8i B cells activated in vitro with increasing concentrations (0.01mg/mL – 10mg/mL) of hapten-antigens at varying hapten density, as well as sHEL and PBS controls. Cultures were supplemented with BAFF (20ng/mL). *(right)* B1-8i receptor internalisation was then measured by flow cytometry using surface NP-PE and λ1 co-stain. Representative FACS plots depict NP and λ1 profile following stimulation with negative controls (C10, HEL) or NP1-RSA stimulation. Datapoints are representative of two independent experiments. **C,** Serum from mice immunized with 100µg OVA/alum or 100µg NP-KLH/alum were harvested at day 8 or day 28 post-immunization, respectively, and added to ELISA plates coated with 10µg/mL OVA, NP3OVA, NP9OVA, NP19OVA or NP25BSA. Each line represents an individual mouse (OVA, n=4; NP-KLH, n=1). Data are from a single experiment.

Supplementary Figure 4. Effects of NUR77 on B cell repertoire and competition, Related to main figure 5.

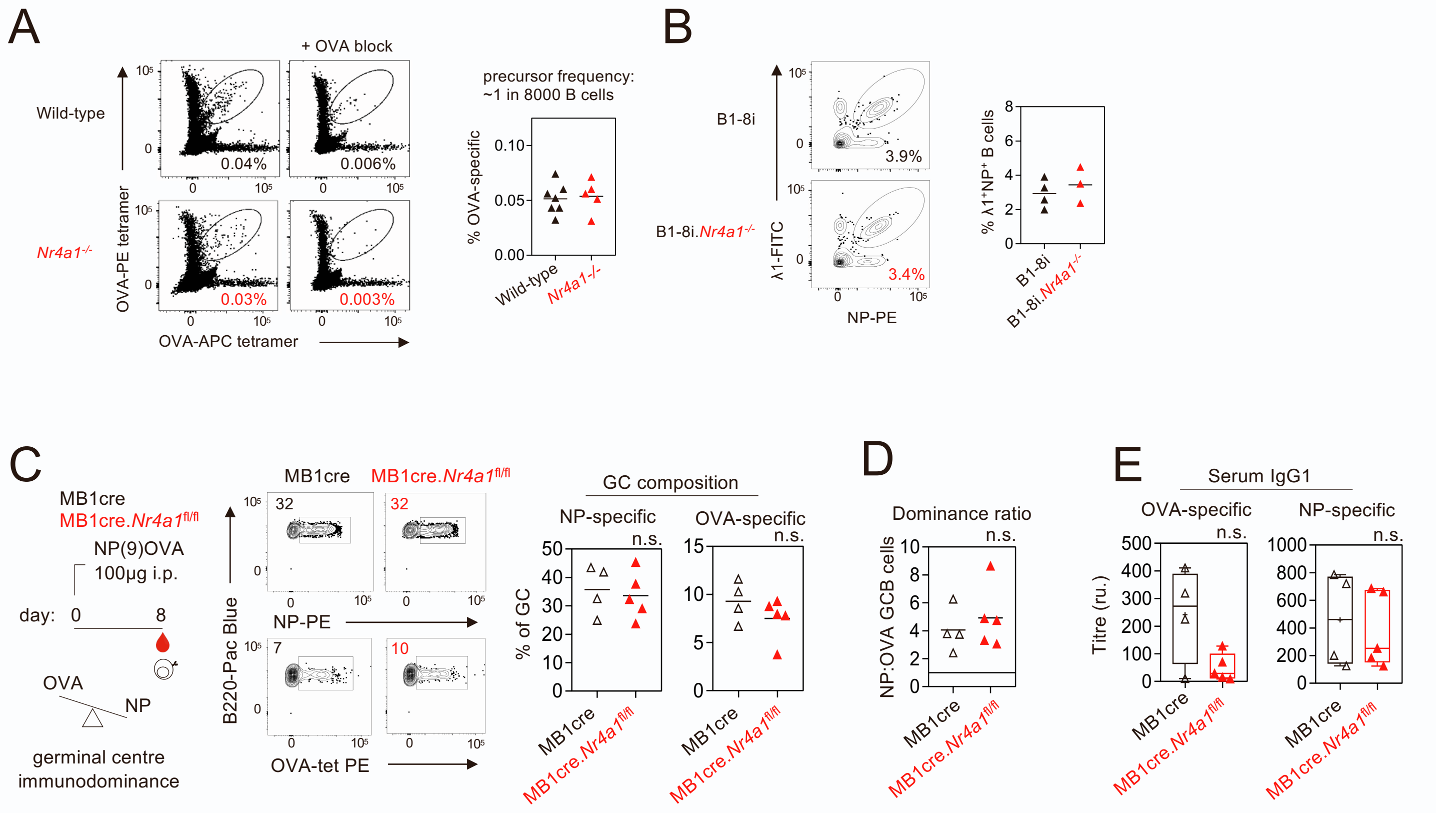

A, *(left)* Representative FACS plots show OVA-specific B cells detected in the spleen by complementary APC and PE-labelled OVA tetramers. As a specificity control, cells were pre-incubated with OVA prior to tetramer staining. *(right)* graph depicts the frequency of mature naive follicular OVA-specific B cells from unimmunized wild-type and *Nr4a1*$^{-/-}$ mice. Precursor frequency within naïve repertoire interpolated as approximately 1:8000 B cells. **B,** *(left)* representative FACS plots showing live NP-specific l1+ splenic B cells in B1-8i *Nr4a1*$^{+/+}$ or *Nr4a1*$^{-/-}$ mice. *(right)* graph depicts frequency of NP-specific splenic B cells from wild-type and *Nr4a1*$^{-/-}$ mice. **C-E,** As in main Figure 5G-I, but using independently generated NP9-OVA immunogen. Data are pooled from two experiments (A, B) or from a single experiment (C-E) and show individual mice. Data were compared by unpaired parametric t-test.

Supplementary Figure 5. Testing GC roles for NUR77 in an alternative model system, Related to main figure 5.

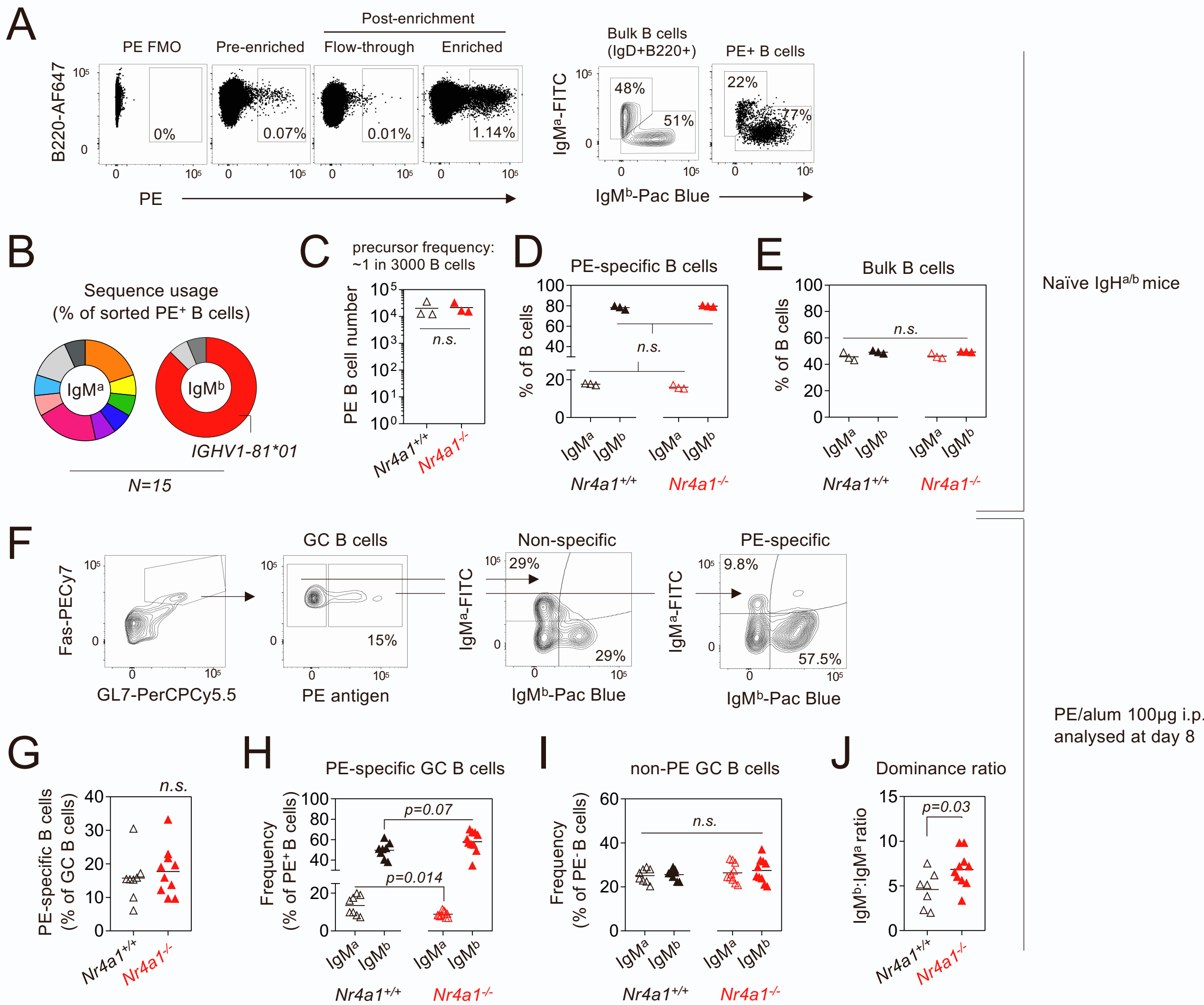

A, Splenocytes from naïve IgH$^{a/b}$ mice were stained with PE and subjected to anti-PE magnetic enrichment. *(left)* Representative FACS plots show sensitivity and specificity of enrichment for PE-binding B cells. *(right)* Representative FACS plots show IgM^a and IgM^b surface BCR expression after gating on either total mature naïve follicular IgD+B220+ B cells or PE-specific B cells. **B,** enriched PE-specific IgM^a and IgM^b B cells were sorted from unimmunized, naïve IgH$^{a/b}$ mice and heavy chains were sequenced. Pie graph shows individual heavy chain usage by IgM^a and IgM^b PE-specific B cells represented as a proportion of all sequences. **C-E,** PE-binding B cells were isolated from naïve repertoire of *Nr4a1*$^{+/+}$ IgH$^{a/b}$ and *Nr4a1*$^{-/-}$ IgH$^{a/b}$ mice following magnetic enrichment as in C. **C,** Graph depicts number of PE-binding B cells isolated from individual naïve mice. **D-E,** Graph depicts relative frequency of IgM^a and IgM^b expression among PE-binding B cells and among total B cells from individual naïve mice, as gated in A. **F-J,** *Nr4a1*$^{+/+}$ IgH$^{a/b}$ and *Nr4a1*$^{-/-}$ IgH$^{a/b}$ mice were immunized with PE/alum (100mg) i.p. and splenic GC were probed 8 days later for PE binding. **F,** Representative FACS plots show gating strategy to identify PE-binding B cells in the GC. **G,** Graph depicts frequency of PE-binding GC B cells as a proportion of total GC B cells. **H,** Graph depicts proportions of PE-binding GC B cells of either IgM^a or IgM^b origin. **I,** As in H, but for non-PE-specific GC B cells. **J,** Graph depicts ratio of IgM^b to IgM^a PE-specific GC B cells. Data are pooled from two independent experiments (A, C-E), from 4 independent experiments (F-J), or from a single experiment (B) and show individual mice. Data were compared by unpaired parametric t-test.
